# Raising Placebo Efficacy in Antidepressant Trials Across Decades Explained by Small-Study Effects: A Meta-Reanalysis

**DOI:** 10.3389/fpsyt.2020.00633

**Published:** 2020-07-28

**Authors:** Lisa Holper

**Affiliations:** Department of Psychiatry, Psychotherapy, and Psychosomatics, University Hospital of Psychiatry, University of Zurich, Zurich, Switzerland

**Keywords:** placebo, antidepressants, small-study effects, time trend, meta-analysis

## Abstract

**Background:**

Recent meta-analyses reported placebo response rate in antidepressant trials to be stable since the 1970s. These meta-analyses however were limited in considering only linear time trends, assessed trial-level covariates based on single-model hypothesis testing only, and did not adjust for small-study effects (SSE), a well-known but not yet formally assessed bias in antidepressant trials.

**Methods:**

This secondary meta-analysis extends previous work by modeling nonlinear time trends, assessing the relative importance of trial-level covariates using a multimodel approach, and rigorously adjusting for SSE. Outcomes were placebo efficacy (continuous), based on the Hamilton Depression Scale, and placebo response rate.

**Results:**

Results suggested that any nonlinear time trends in both placebo efficacy (continuous) and response rate were best explained by SSE. Adjusting for SSE revealed a significant gradual increase in placebo efficacy (continuous) from 1979 to 2014. A similar observation was made for placebo response rate, but did not reach significance due higher susceptibility to SSE. By contrast, trial-level covariates alone were found to be insufficient in explaining time trends.

**Conclusion:**

The present findings contribute to the ongoing debate on antidepressant placebo outcomes and highlight the need to adjust for bias introduced by SSE. The results are of clinical relevance because SSE may affect the evaluation of success or failure in antidepressant trials.

## Highlights

- Placebo time trends in antidepressant trials were examined between 1979 and 2014.
- Placebo efficacy (continuous) was found to gradually linearly increase across decades.
- Placebo response rate did not reach significance due to large small-study effects.
- Small-study effects explained large proportions of heterogeneity.
- Trial-level covariates did not sufficiently explain heterogeneity.
- Small-study effects may be considered when evaluating success or failure in antidepressant trials.

## Introduction

The term placebo effect is commonly used to designate symptom relief due to a non-pharmacological intervention that cannot be attributed to drug properties, and is therefore thought to reflect patient expectations regarding that intervention (1). While it is well-known that individuals receiving placebo in antidepressant trials in major depressive disorder (MDD) can show substantial improvement (2), there has been an ongoing debate whether this placebo effect has been raising since the 1970s (3–10). This debate is of clinical relevance because placebo effects have been suspected to contribute to a decline in antidepressant treatment effects, resulting in so-called failed antidepressant trials (3, 5, 6, 11–13).

Two recent meta-analyses conducted by Furukawa et al. (14, 15) disproved this hypothesis. The authors, who meta-analytically examined N = 252 trials conducted between 1979 and 2016, reported that placebo response rate in antidepressant trials have remained stable from 1979 to 2016. Any selective improvement in placebo response was suggested to result from trials conducted before 1991, at which year a structural break was found, after which placebo response rate was reported to be (14). The same conclusion was made by the authors based on a smaller dataset comprising N = 98 trials conducted between 1985 and 2012 (15). To assess the effects of study year, Furukawa et al. (14, 15) splitted the data and computed linear regressions separately before and after the observed structural break at 1991. Splitting data for regression is however not advisable to account for the likely nonlinearity underlying the structural break. The present analysis therefore aimed to model the nonlinear time trend to regress across the entire time span from 1979 to 2016. For this purpose, the present analysis implemented restricted cubic splines (RCS) (16), which are a powerful technique for modeling nonlinear relationships using linear regression models.

Furukawa et al. (14, 15) further suggested that any potential increase in placebo response rate before 1991 may be explained by smaller mostly older studies showing larger treatment effects than bigger studies. This phenomenon, known as small-study effect (SSE) (17, 18) describes the association between sample size and effect size in meta-analyses. To account for SSE, Furukawa et al. (14, 15) adjusted the regression for sample size (defined as the number of patients randomized). When adjusting for SSE, however, there is actually a choice between a function of sample size (or its inverse version) or a function of study precision (variance, standard error, or their inverse versions), depending on which one is more closely related to the sources of SSE. One may argue that a function of sample size is the better covariate because it does not experience measurement error or structural correlation. However, as extensively studied by Moreno (19) a function of precision is preferable since the sources of SSE have increasingly been attributed to reasons such as publication bias, outcome reporting bias, or clinical heterogeneity (20–22). A function of study precision is therefore thought to be more informative (23). The present analysis therefore aimed to account for possible SSE by allowing placebo outcomes to depend on the standard error (24, 25).

Moreover, Furukawa et al. (14, 15) suggested that the potential increase in placebo response rate may also be explained by changes in study designs across decades. The authors therefore adjusted for the trial-level covariates study center, study dosing schedule, and study length, which were found to be significant. In particular, increasing placebo response rate were suggested to be associated with shorter, single-centered trials using flexible dose regimes before 1991 compared to longer, multi-centered trails using fixed dose regimens mostly conducted after 1991. The interpretation of these assumptions however remained unclear; for example, it remained unclear why multi- versus single-centered trials or fixed versus flexible dose regimes would increase placebo effect sizes. The present analysis therefore hypothesized that these trial-level covariate effects are mediated effects (26) arising from insufficient adjustment for SSE across decades. To assess the relative importance of trial-level covariates effects before and after adjustment for SSE, multimodel inference was conducted (27). Multimodel inference is an information theoretic approach proposed as an alternative to traditional single-model hypothesis testing as applied by Furukawa et al. (14, 15). Multimodel inference examines several competing hypotheses (models) simultaneously to identify the best set of models *via* information criteria such as the Akaike’s information criterion (28), and is thus thought to provide more robust covariate estimates (27).

Last, Furukawa et al. (14, 15) estimated placebo response rate (defined as ≥50% reduction on the HAMD) based on the proportion of responders *within* placebo groups. In the second meta-analysis (15) the authors additionally assessed the original continuous outcome, i.e., symptom reduction (29) based on the Hamilton Depression Scale (HAMD) (30), from which the binary outcome (response rate) is derived through dichotomization. The continuous outcome was estimated based on the drug-placebo difference *between* drug and placebo groups. The authors did, however, not consider symptom reduction *within* placebo groups, which would be the logical equivalent to placebo response rate. The present analysis therefore aimed to extend previous work by examining symptom reduction within placebo groups estimated based on the pre-post change on the HAMD within placebo groups, hereinafter referred to as efficacy (continuous).

Together, the methodological approach presented here aimed to support the ongoing debate on placebo outcomes across decades by considering nonlinear time trends, adjusting for SSE, assessing the relative importance of trial-level covariates, and comparing the binary with the original continuous placebo outcome. The results are expected to inform clinical decision making whether time trends may require consideration when evaluating success or failure in antidepressant trials in MDD.

## Materials and Methods

### Data Sources

A total of 308 randomized placebo-controlled trials (240 published studies, 68 unpublished studies) were identified conducted between 1979 and 2016. Three hundred four trials constituted all the placebo-controlled trials provided in the GRISELDA dataset by Cipriani et al. (31) and four additional trials were provided by Furukawa et al. (14). For all studies, information on the year of completion was extracted from the literature, if available. For 30 studies, missing covariate values were extracted from the literature. A PRISMA (Preferred Reporting Items for Systematic Reviews and Meta-Analyses) flow-chart detailing the study selection process is given in the supplementary appendix (**Supplementary S1**).

Covariate study year was defined as study year of completion, study year of publication, or year of drug approval from the FDA (US Food and Drug Administration), where available in this order; preference was given to study year of completion, because unpublished trials, by definition, have no year of publication (14). The resulting study year range was 1979–2014. Other trial-level covariates were study center (multi- versus single-center), study dosing schedule (flexible versus fixed dose), study length (range 4–12 weeks), and study size (sample size, number of patients randomized) (14).

Primary continuous outcome was efficacy (continuous) (pre-post change on the HAMD, i.e., endpoint HAMD or, if not reported, change from baseline HAMD, as provided in the GRISELDA dataset (31), N = 259 trials) within placebo groups, estimated as log-transformed change score. Primary binary outcome was response rate (≥50% reduction on the HAMD, N = 277 trials), estimated as log-transformed proportions of responders within placebo groups. Remission rate was not assessed because different cut-offs (<7 or <8 total reduction on the HAMD) were used for aggregation, which can be problematic when analyzing only within groups.

### Structural Break Analysis

To assess potential breaks across study year, a structural break analysis was conducted using the breakpoints command in the strucchange package (32) in R (33). To capture potential structural breaks in the meta-regression, the covariate study year was modeled using RCS (16) by use of the command rcs in the rms package (34). RCS were constructed using three knots with the middle knots set at the break date, and the first and third knots set at the 10*^th^* and 90*^th^* percentile of study year, i.e., (1986, break date, 2010).

### Adjustment for Small-Study Effects

To adjust for SSE, limit meta-analysis was conducted using the metasens package (35) (**Supplementary S4**). Limit meta-analysis is a regression approach based on an extended random-effects model that takes account of possible SSE by allowing trial estimates to depend on the standard error (24, 25, 36). The resulting adjusted (“shrunken”) trial estimates obtained from the limit meta-analysis were then used for further analysis. Heterogeneity that remains after SSE are accounted for can be quantified by the heterogeneity statistic G^2^ (24, 25).

To graphically visualize potential SSE, the power of individual studies was computed based on its standard errors using a two-sided Wald test, together with a test assessing potential excess of formally significant trials in relation to the power (37) as implemented in the metaviz package (38). The test itself does not consider SSE but can have similar sources.

### Multimodel Inference

Multimodel inference was conducted using the glmulti package (39) that provides the necessary functionality for multimodel averaging using an information-theoretic approach. An extensive model comparison was conducted considering all possible covariate combinations. Multimodel inference was performed both before and after adjustment for SSE based on the limit meta-analysis. The only constraint was that the nonlinear component of study year was only included in the presence of the linear component, since the former alone is uninterpretable. Together this resulted in N = 47 models. Model weighting was based on the corrected Akaike information criterion (AICc), with each model being weighted based on relative AICc evidence (39). Multimodel averaged covariate estimates were computed across the top model sets for each outcome, defined as the top-ranked models summing up to 95% AICc evidence weight (27, 40). Models were fitted using the rma function in the metafor package (41). Heterogeneity was estimated based on the method of moment (42) and reported in terms of τ^2^ and I^2^.

## Results

### Structural Break Analysis

Structural break analysis revealed no break date for placebo efficacy (continuous) (F = 3·09, p = 0·139), suggesting a gradual increasing trend from 1979 to 2014. By contrast, a break date was found at 1990 for placebo response (F = 11·45, p < 0·001), suggesting a steep nonlinear increase around 1990; in line with Furukawa et al. (14) (see **Supplementary S2** for illustration of the structural break analysis). To assess the potential nonlinear trend across decades, the break date at 1990 was used to construct the nonlinear component of the covariate study year used in the multimodel inference (see **Supplementary S3** for sensitivity analysis on the nonlinear component).

### Multimodel Averaged Covariate Effects

**Figure 1:**
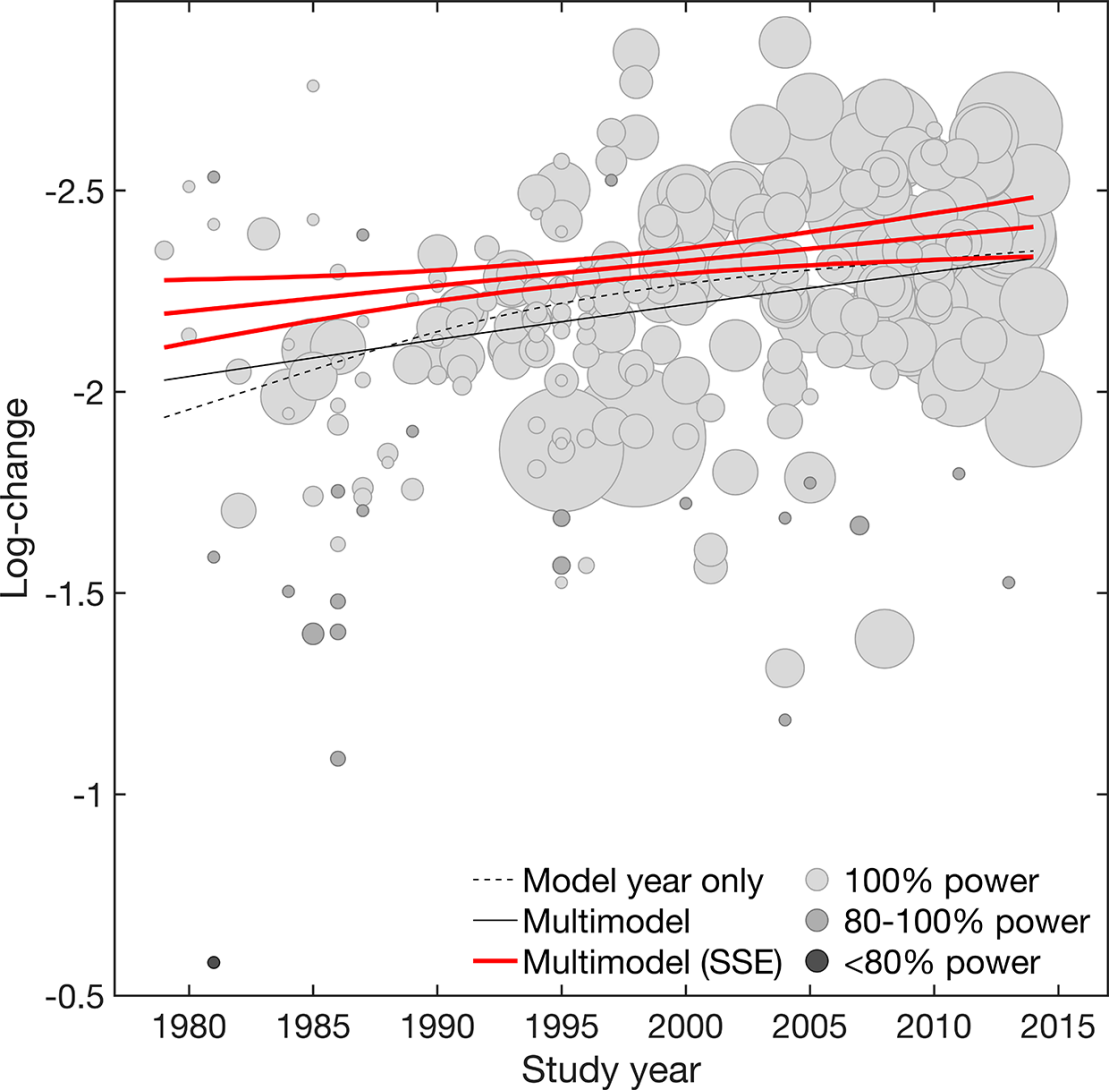
Efficacy (continuous). Meta-regression plot illustrating the effect of study year on placebo efficacy (continuous) for the model adjusted for year only and the multimodel average considering both main and interaction effects, before and after accounting for small-study effects (SSE). Values larger on the log-transformed change score scale indicate increase in efficacy (continuous). Circle size is proportional to study size. Circle color is proportional to the power of individual studies (100% high power [white], 100-80% adequate power [light gray], <80% low power [dark gray]). Slopes are illustrated at the means of all covariates.

**Figure 2:**
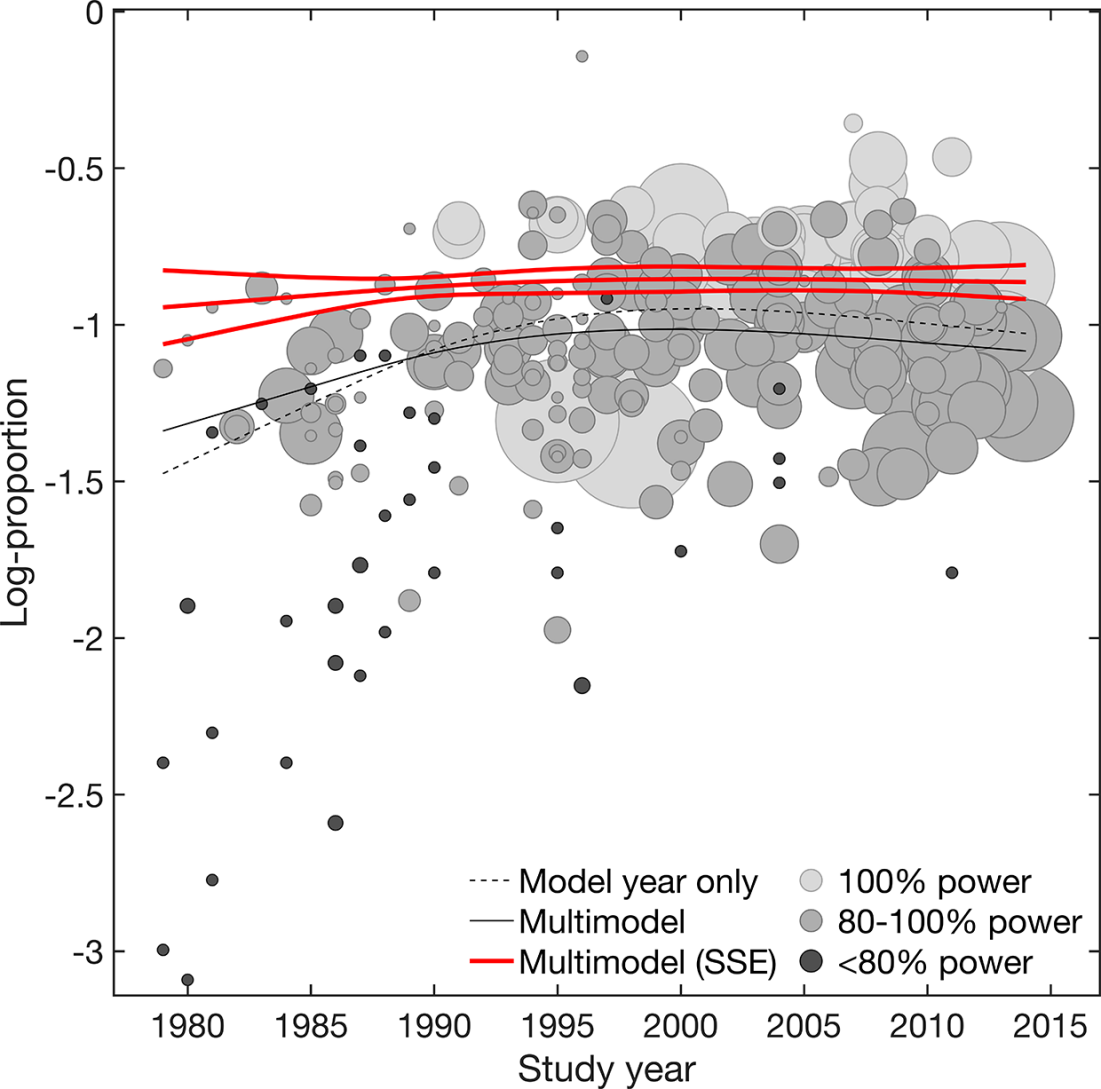
Response rate. Meta-regression plot illustrating the effect of study year on placebo response rate. Shown are the model adjusted for year only and the multimodel average considering both main and interaction effects, before and after accounting for small-study effects (SSE). Values smaller on the log-transformed proportion scale indicate increase in response rate. Circle size is proportional to study size. Circle color is proportional to the power of individual studies (100% high power [white], 100-80% adequate power [light gray], <80% low power [dark gray]). Slopes are illustrated at the means of all covariates.

Overall, results suggested both placebo efficacy (continuous) and response rate to be affected by SSE. Adjustment for SSE weakened the effect of study year in both outcomes. However, the linear effect of study year indicated by the structural break analysis remained significant for efficacy (continuous) [linear β = −0·07 (−0·12 to −0·02) 95% CI; nonlinear β = 0·00 (−0·02 to 0·02) 95% CI] (**Table 1**, **Figure 1**). This supported a gradual increase in placebo efficacy (continuous) across decades from 1979 to 2014, that is not explained by SSE. By contrast, both linear and nonlinear components of study year became insignificant for placebo response due to wide CIs [linear β = 0·07 (−0Â·05 to 0·18) 95% CI; nonlinear β = −0·05 (−0·14 to 0·05) 95% CI] (**Table 1**, **Figure 2**). Together, this suggested that the binary outcome was more affected by SSE, with lower study precision observed in older and smaller studies before 1991 compared to studies conducted after 1990. Among the other trial-level covariates only study center revealed significant effects in both outcomes. However, supporting our hypothesis of possible mediated effects, any effects of study center became nearly zero after adjusting for SSE (**Table 2**). None of the remaining trial-level covariates were found to have significant effects (see **Supplementary S5** for details on relative covariate importance). Together, this suggested that trial-level covariates alone were insufficient in explaining placebo outcomes.

**Table 1:** Multimodel averaged covariate estimates.

	Efficacy (cont.)		Response	
Model year only	β (95% CI)		β (95% CI)	
Year linear	−0.21 (−0.34 to −0.07)		0.39 (0.25 to 0.53)	
Year nonlinear	0.09 (−0.03 to 0.21)		−00.28 (−0.41 to −00.16)	
**Multimodel**	**β (95% CI)**	**W**	**β (95% CI)**	**W**
Year linear	−0.10 (−0.14 to −00.05)	1.0	0.26 (0.12 to 0.40)	1.0
Year nonlinear	0.01 (−0.01 to 0.02)	0.1	−00.19 (−0.32 to −00.07)	1.0
Center	−0.20 (−0.30 to −00.10)	1.0	0.28 (0.18 to 0.37)	1.0
Dosing	−0.01 (−0.06 to 0.03)	0.5	0.01 (−0.03 to 0.05)	0.3
Length	0.00 (−0.01 to 0.01)	0.3	0.02 (−0.02 to 0.06)	0.7
Size	0.00 (−0.01 to 0.01)	0.2	−00.01 (−0.03 to 0.02)	0.4
**Multimodel (SSE)**	**β (95% CI)**	**W**	**β (95% CI)**	**W**
Year linear	−00.07 (−0.12 to −00.02)	1.0	0.07 (−0.05 to 0.18)	0.8
Year nonlinear	0.00 (−0.02 to 0.02)	0.2	−00.05 (−0.14 to 0.05)	0.6
Center	−00.00 (−0.02 to 0.01)	0.1	0.01 (−0.03 to 0.05)	0.3
Dosing	0.00 (−0.02 to 0.02)	0.3	0.00 (−0.01 to 0.02)	0.2
Length	0.00 (−0.01 to 0.01)	0.3	0.02 (−0.01 to 0.05)	0.8
Size	0.02 (−0.01 to 0.05)	0.7	−00.05 (−0.08 to 0.02)	1.0

Listed are standardized covariate estimates (95% confidence intervals, CI) for the model adjusted for year only and the multimodel average before and after accounting for small-study effects (SSE). The relative covariate importance is given in terms of AICc evidence weights (W), with larger values indicting greater importance, whereas values near zero indicate that there is little or no evidence that the given covariate of interest explains variation in the corresponding outcomes. Significant effects (p < 0·05) are highlighted (red).

**Table 2:** Multimodel averaged heterogeneity statistics.

Efficacy (cont.)	τ^2^(%)	I^2^	G^2^
Model year only	0.0456 (−13%)	88%	
Multimodel	0.0449 (−15%)	88%	
Multimodel (SSE)	0.0282 (−46%)	82%	3%
**Response**	**τ^2^(%)**	**I^2^**	**G^2^**
Model year only	0.0386 (−12%)	71%	
Multimodel	0.0377 (−13%)	71%	
Multimodel (SSE)	0.0141 (−68%)	47%	24%

Listed are heterogeneity statistics for the model adjusted for year only and the multimodel average considering both main and interaction effects, before and after accounting for small-study effects (SSE), in terms of τ^2^ [with the percentage reduction (%) compared to the unadjusted model] and I^2^. Listed is also the heterogeneity statistic G^2^ (scaled 0–100%) derived from the limit meta-analysis, interpreted as the proportion of unexplained heterogeneity after accounting for SSE.

To graphically visualize the suggested SSE, the power of individual studies was illustrated in **Figures 1** and **2**. The power distributions show that most of the trials estimates for efficacy (continuous) were high-powered (0Â·4% low power, 8% adequate power, 91% high power) with no excess of significant trials (p = 0·236), whereas placebo response rate was underpowered (13% low power, 70% adequate power, 17% high power) with an excess of significant trials (p = 0·001) (see **Supplementary S4** for funnel. plots derived from limit meta-analysis compared to power-enhanced funnel plots). Together, this supported the assumption that placebo response rate was more affected by SSE than placebo efficacy (continuous).

### Multimodel Averaged Heterogeneity

Since, it is well-known that I^2^ has undue reliance due to its dependence on precision (43, 44), it is an improper measure when comparing continuous and binary outcomes because the former result in systematically higher I^2^ (45). τ^2^, rather than I^2^, is therefore suggested the appropriate measure for this purpose as it is insensitive to study precision (43) (**Table 2**). Comparing between outcomes suggested an overall equal reduction in heterogeneity (efficacy (continuous) = −13%, response rate = −12%) when SSE were not accounted for. However, when accounting for SSE, a much larger reduction in heterogeneity was observed for response rate (−70%) compared to efficacy (continuous) (−37%). Again, this supported the assumption of larger SSE in the binary compared to the continuous outcome.

Heterogeneity was also assessed in terms of G^2^ derived from the limit meta-analysis. G^2^ represents heterogeneity that remains after SSE are accounted for. Likewise I^2^, G^2^ is determined on a percentage scale, but not dependent on study precision and thus appropriate for comparison between outcomes (25, 43). Whereas G^2^ was almost zero for placebo efficacy (continuous) (G^2^ = 2%), it was G^2^ = 24% for placebo response rate. This indicated that efficacy (continuous) approximated the case of G^2^ = 0 while > 0, that is, there is not much other heterogeneity apart from that due to SSE (to which is sensitive but not G^2^) (25) (**Table 2**, see **Supplementary S4** for details on the limi meta-analysis). Together, this suggested that efficacy (continuous) was almost fully explained by SSE, whereas placebo response rate remained with some unexplained heterogeneity even after adjustment for SSE.

## Discussion

Selective improvements in placebo effects can significantly affect the success or failure of antidepressant trials. Declining antidepressant efficacy across decades has been previously suggested due to increasing placebo efficacy, resulting in so-called failed antidepressant trials (3, 5, 6, 11–13). Study year has been suggested to be the second greatest effect modifier in antidepressant efficacy as reported by Cipriani et al. (31), but this itself does not inform about why outcomes are heterogeneous across years. It is therefore of clinical relevance to explore the reasons of potentially increasing placebo effects across decades.

Furukawa et al. (14, 15) suggested that any potential secular changes in placebo outcomes may be explained by changes in study designs corresponding to differences in study centers, study dosing schedule, study length, or study size. The present findings rather suggest that small-study effects drive most of the changes in placebo outcomes across decades, due to older mostly smaller antidepressant trials being greatly underpowered. This assumption is in line with previous work, suggesting fundamental flaws in antidepressant trials due to underpowered effects sizes and a lack of precision in depression outcome measurements for the past 40 years (46).

The present findings show that adjusting for these small-study bias largely reduces heterogeneity and removes any nonlinear effects of study year. Still, a remaining linear trend is suggested for efficacy (continuous) gradually increasing from 1979 to 2014, that is not explained by small-study effects. This increasing trend does not seem to follow a structural break as suggested by Furukawa et al. (14, 15), again disproving any underlying nonlinearity. Considering the low heterogeneity that remained after small-study effects were accounted for (G^2^ = 3%), suggests that further bias adjustments may not essentially change these findings.

By contrast, trial-level covariates alone were found to be insufficient in explaining placebo time trends and may even led to misleading conclusions by Furukawa et al. (14, 15). The present work rather suggests that at least part of the trial-level covariate effects may be mediated by small-study effects without having own effects. An example is the effect of study center that was found to be insignificant after adjusting for small-study effects. This is likely a result of the close relation between secular changes from single-centered trials with small sample size (and thus low study precision) in older trials, versus more multi-centered trials with larger sample sizes (and thus higher study precision) in more recent trials. This illustrates the importance of adjusting for small-study effects in order to derive reliable trial-level covariate effects in antidepressant trials.

The observed differences between the continuous and binary outcomes are likely a result of the dichotomization of the original HAMD scale. In clinical practice, dichotomization is sometimes justified to label groups of individuals with diagnostic or therapeutic attributes (47, 48). However, methodologists have advised against the use of dichotomization because it reduces statistical power and inflates effect sizes (49–52). To derive binary constructs, the quantitative HAMD scale is dichotomized along arbitrary cut-off scores (49, 51, 53, 54). This can create artificial boundaries, were patients just below and above the cut-off fall into different binary (49). For example, a responder, who drops 50% from 40 to 20 on the HAMD can still be quite depressed, while someone who drops from 20 to 10 is almost in remission. Compared with efficacy (continuous), response rates are thus less precise since most of the information distinguishing patients on the original scale is lost (52).

These aspects are even more problematic considering small sample sizes, because the reduction in statistical power consequently requires larger sample sizes in binary outcomes to be adequately powered (55). Although, there are no clear sample size requirements for meta-analyses, previous work showed that small trials (e.g., fewer than 50 patients) can produce 10–48% larger binary estimates than larger trials (56). Most trials completed before 1990 had on average less than 50 patients. As a consequence, the power of the binary outcome was reduced before 1990. This is because the precision of the binary outcome is statistically dependent on its effect estimate. This dependence induces a well-known asymmetry in funnel plots, a sort of mathematical artifact (57, 58), which also explains the steep nonlinear trend suggested by the break analysis. As a consequence, placebo response rate showed (59). The present findings thus provide another example that response rate in antidepressant trials may generally be avoided due to low statistical power and spuriously inflated effect sizes. The continuous outcome may be preferred when available, which is viewed as a more favorable endpoint as it is less susceptible to small-study effects.

Limiting the present analysis is the fact that the described phenomena of underpowered effect sizes and small-study effects can cause similar results besides having different reasons. Interpretation should therefore be cautious, given that it is not possible to separate the different mechanisms of bias (37, 60). Another possible and probably the most well-known reason for small-study effects is publication bias, which occurs when the chance of smaller studies being published is increased when having significant positive results, compared to larger studies which may be accepted and published regardless. Notably, publication bias can also arise from time-lag effects, resulting from the variability in the time it takes to complete and publish a study (61). Weak or negative results have been shown to take approximately two to three more years to be published compared to stronger and positive results (62, 63). To reduce the implications of potential time-lag effects (and to allow for the inclusion of non-published studies), the present analysis therefore synthesized results across completion years, if available. Other well-known causes of small-study effects are outcome selection bias, where only favorable outcomes are selectively reported (20, 64, 65), and clinical heterogeneity, e.g., patients in smaller studies may have been selected so that a favorable outcome is to be expected (59), both of which are not addressed in the present analysis.

Another limitation of the present analysis is that it did not separately address risk of bias (RoB) and how this relates to SSE, because it is difficult to relate RoB only to placebo outcomes without consideration of the drug-related RoB. The funnel plots and Egger’s test as detailed in the supplementary appendix (**Supplementary S4**), however, give at least some quantification of RoB related to the presence of possible publication bias. For a detailed assessment of RoB of the included studies it is referred to Cipriani et al. cipriani (31).

Further research may be required to explain the reasons for the observed increase in placebo efficacy (continuous) across decades. Earlier meta-analyses considered several other trial-level or patient characteristics, such as the use of placebo run-in, probability of being allocated to placebo, number of trial arms, publication status, co-medication, country region, primary versus secondary care, inpatient versus outpatient settings, age, sex, and baseline depression severity (3, 5, 7, 9, 14, 66–70); none of which consistently explained placebo outcomes. Based on the extensive previous work, it therefore seems unlikely that other trial-level or patient characteristics can fully explain the present observations. One might therefore also consider factors other than measurable covariates. For example, it has been suggested that marketing has led to an increased public perception that antidepressants are effective. This may increase the consumer demand for the thus advertised antidepressants, and may create conditioned responses and expectations that can produce a placebo effect similar to that when the medication is taken (71, 72). Others have argued that inter-rater and intra-rater variabilities contributed to this phenomenon (73–75). Again, others suggested that the issue of unblinded outcome-assessors in double-blind trials has led to worse placebo outcomes in older compared to more recent trials due to the older drugs’ marked side effects compared to newer generation antidepressants (76). All these aspects may also be related to overall higher standards in trial conductance by pharmaceutical companies in more recent trials, which not only significantly increased sample sizes, but may have also increased expectations in placebo receiving individuals (46).

In conclusion, there has been an ongoing debate how placebo outcomes in antidepressant trials can be explained across decades. The present analysis aimed to contribute to the debate suggesting that secular changes in placebo outcomes are best explained by small-study effects, rather than by trial-level covariates. Further research may be required to adjust the corresponding antidepressant treatment effects for small-study effects, to account for increase in placebo efficacy (continuous). This is of clinical relevance to evaluate success or failure in antidepressant trials.

## Data Availability Statement

All datasets presented in this study are included in the article/**Supplementary Material**.

## Author Contributions

The author confirms being the sole contributor of this work and has approved it for publication.

## Funding

This work was funded by the Swiss National Science Foundation (SNSF).

## Conflict of Interest

The author declares that the research was conducted in the absence of any commercial or financial relationships that could be construed as a potential conflict of interest.
