# Supplementary material for: Raising Placebo Efficacy in Antidepressant Trials Across Decades Explained by Small-Study Effects: A Meta-Reanalysis
Source: Front Psychiatry. 2020 Jul 28;11:633. doi: 10.3389/fpsyt.2020.00633 (PMC7399231; doi:10.3389/fpsyt.2020.00633)
Supplement: Supplementary file 1 [file DataSheet_1.pdf]

Supplementary Material: Raising placebo efficacy in antidepressant trials across decades explained by small-study effects: a meta-reanalysis

1 LIST OF IDENTIFIED STUDIES (N = 308)

1. **AK1102365**
`https://www.gsk-clinicalstudyregister.com/study/AK1102365#ps`
2. **Alexopoulos 2004 (poster SCT-MD-27)**
`www.ncbi.nlm.nih.gov/pubmedhealth/PMH0016624/bin/app17c_et6.pdf`
3. **Alvarez 2012 (11492A, NCT00839423)**
Alvarez E, Perez V, Dragheim M, Loft H, Artigas F. A double-blind, randomized, placebo-controlled, active reference study of Lu AA21004 in patients with major depressive disorder. Int J Neuropsychopharmacol. 2012;15(5):589-600. `https://clinicaltrials.gov/ct2/show/NCT00839423`
4. **Amin 1984**
Amin MM, Ananth JV, Coleman BS, Darcourt G, Farkas T, Goldstein B, Lapierre YD, Paykel E, Wakelin JS. Fluvoxamine: antidepressant effects confirmed in a placebo-controlled international study. Clinical Neuropharmacology 1984;7(suppl 1):580-581.
5. **Amsterdam 1986**
Amsterdam JD, Case WG, Csanalosi E, Singer M, Rickels K. A double-blind comparative trial of zimelidine, amitriptyline, and placebo in patients with mixed anxiety and depression. Pharmacopsychiatry. 1986 May;19(3):115-9.
6. **Asnis 2013 (LVM-MD-01, NCT00969709)**
Asnis GM, Bose A, Gommoll CP, Chen C, Greenberg WM. Efficacy and safety of levomilnacipran sustained release 40 mg, 80 mg, or 120 mg in major depressive disorder: a phase 3, randomized, double-blind, placebo-controlled study. J Clin Psychiatry. 2013 Mar;74(3):242-8. `https://clinicaltrials.gov/ct2/show/NCT00969709`
7. **Bakish 1992**
Bakish D, Bradwejn J, Nair N, McClure J, Remick R, Bulger L. A comparison of moclobemide, amitriptyline and placebo in depression: a Canadian multicentre study. Psychopharmacology (Berl). 1992;106 Suppl:S98-101.
8. **Bakish 2014 (LVM-MD-10, NCT01377194)**
Bakish D, Bose A, Gommoll C, Chen C, Nunez R, Greenberg WM, Liebowitz M, Khan A. Levomilnacipran ER 40 mg and 80 mg in patients with major depressive disorder: a phase III, randomized, double-blind, fixed-dose, placebo-controlled study. J Psychiatry Neurosci. 2014 Jan;39(1):40-9. `https://clinicaltrials.gov/ct2/show/NCT01377194`
9. **Baldwin 2012 (11984A, NCT00635219)**
Baldwin DS, Loft H, Dragheim M. A randomised, double-blind, placebo controlled, duloxetine-referenced, fixed-dose study of three dosages of Lu AA21004 in acute treatment of major depressive disorder (MDD). Eur Neuropsychopharmacol. 2012 Jul;22(7):482-91. `https://clinicaltrials.gov/ct2/show/NCT00635219`
10. **Ban 1998 (Study 008)**
Ban TA, Gaszner P, Aguglia E, Vergara L. Clinical Efficacy of Reboxetine: a Comparative Study with Desipramine, with Methodological Considerations. Human Psychopharmacology 1998;13: S29-S39. `https://www.iqwig.de/de/projekte-ergebnisse/studieninformationen-zu-reboxetin.3304.html`
11. **Barber 2011**
Barber JP, Barrett MS, Gallop R, Rynn MA, Rickels K. Short-term dynamic psychotherapy versus pharmacotherapy for major depressive disorder: a randomized, placebo-controlled trial. J Clin Psychiatry. 2012 Jan;73(1):66-73.
12. **Bhatia 1991**

Bhatia SC, Hsieh HH, Theesen KA, Townley RG, Andersen JM, Weiss S, Agrawal DK. Platelet alpha-2 adrenoreceptor activity pre-treatment and post-treatment in major depressive disorder with melancholia. Res Commun Chem Pathol Pharmacol. 1991 Oct;74(1):47-57.

13. **Binnemann 2008 (NCT00143091)**
Binneman B, Feltner D, Kolluri S, Shi Y, Qiu R, Stiger T. A 6-week randomized, placebo-controlled trial of CP-316,311 (a selective CRH1 antagonist) in the treatment of major depression. Am J Psychiatry. 2008;165(5):617-20.

14. **Bjerkenstedt 2005**
Bjerkenstedt L1, Edman GV, Alken RG, Mannel M. Hypericum extract LI 160 and fluoxetine in mild to moderate depression: a randomized, placebo-controlled multi-center study in outpatients. Eur Arch Psychiatry Clin Neurosci. 2005 Feb;255(1):40-7.

15. **Bosc 1997a (Study 014 – Andreoli 2002)**
Andreoli V, Caillard V, Deo RS, Rybakowski JK, Versiani M. Reboxetine, a new noradrenaline selective antidepressant, is at least as effective as fluoxetine in the treatment of depression. J Clin Psychopharm 2002; 22: 393–99.

16. **Bose 2008 (SCT-MD-13)**
Bose A, Li D, Gandhi C. Escitalopram in the Acute Treatment of Depressed Patients Aged 60 Years or Older. Am J Geriatric Psychiatry. 2008 Jan;16(1):14-20.

17. **Boulenger 2014 (13267A, NCT01140906)**
Boulenger JP, Loft H, Olsen CK. Efficacy and safety of vortioxetine (Lu AA21004), 15 and 20 mg/day: a randomized, double-blind, placebo-controlled, duloxetine-referenced study in the acute treatment of adult patients with major depressive disorder. Int Clin Psychopharmacol. 2014;29(3):138-49.
`https://clinicaltrials.gov/ct2/show/NCT01140906`

18. **Boyer 2008 (Study 333, NCT00300378, EUCTR2005-005463-28, 3151A1-333-EU)**
Boyer P, Montgomery S, Lepola U, Germain JM, Brisard C, Ganguly R, Padmanabhan SK, Tourian KA. Efficacy, safety, and tolerability of fixed-dose desvenlafaxine 50 and 100 mg/day for major depressive disorder in a placebo-controlled trial. Int Clin Psychopharmacol. 2008;23(5):243-53.
`https://clinicaltrials.gov/ct2/show/NCT00300378`

19. **Bremner 1995 (MIR 003-022 - FDA)**
Bremner JD. A double-blind comparison of Org 3770, amitriptyline, and placebo in major depression. J Clin Psychiatry. 1995 Nov;56(11):519-25.

20. **Brown 1986**
Brown WA, Arato M, Shrivastava R. Pituitary-adrenocortical hyperfunction and intolerance to fluvoxamine, a selective serotonin uptake inhibitor. Am J Psychiatry. 1986 Jan;143(1):88-90.

21. **Brunoni 2012**
Brunoni AR, Valiengo L, Baccaro A, Zanão TA, de Oliveira JF, Goulart A, Boggio PS, Lotufo PA, Benseñor IM, Fregni F. The sertraline vs. electrical current therapy for treating depression clinical study: results from a factorial, randomized, controlled trial. JAMA Psychiatry. 2013;70(4):383-91.

22. **Buchsbaum 1997**
Buchsbaum MS, Wu J, Siegel BV, Hackett E, Trenary M, Abel L, Reynolds C. Effect of sertraline on regional metabolic rate in patients with affective disorder. Biol Psychiatry. 1997 Jan 1;41(1):15-22.

23. **Burke 2002 (SCT-MD-01)**
Burke WJ, Gergel I, Bose A. Fixed-dose trial of the single isomer SSRI escitalopram in depressed outpatients. J Clin Psychiatry. 2002 Apr;63(4):331-6

24. **Byerley 1988**

Byerley WF, Reimherr FW, Wood DR, Grosser BI. Fluoxetine, a selective serotonin uptake inhibitor, for the treatment of outpatients with major depression. J Clin Psychopharmacol. 1988;8(2):112-5.

25. **CAGO178A2303 (NCT00463242)**
https://clinicaltrials.gov/ct2/show/record/NCT00463242

26. **Carman 1991**
Carman JS, Ahdieh H, Wyatt-Knowles E, Warga E, Panagides J. A controlled study of mianserin in moderately to severely depressed outpatients. Psychopharmacol Bull. 1991;27(2):135-9.

27. **Cassano 1986**
Cassano GB, Conti L, Massimetti G, Mengali F, Waekelin JS, Levine J. Use of a standardized documentation system (BLIPS/BDP) in the conduct of a multicenter international trial comparing fluvoxamine, imipramine, and placebo. Psychopharmacol Bull. 1986;22(1):52-8.

28. **CL3-20098-022**
http://www.ema.europa.eu/docs/en_GB/document_library/EPAR_-_Public_assessment_report/human/000916/WC500038315.pdf

29. **CL3-20098-023**
http://www.ema.europa.eu/docs/en_GB/document_library/EPAR_-_Public_assessment_report/human/000915/WC500046226.pdf

30. **CL3-20098-024**
http://www.ema.europa.eu/docs/en_GB/document_library/EPAR_-_Public_assessment_report/human/000916/WC500038315.pdf

31. **CL3-20098-026**
http://www.ema.europa.eu/docs/en_GB/document_library/EPAR_-_Public_assessment_report/human/000916/WC500038315.pdf

32. **CL3-20098-070**
https://www.clinicaltrialsregister.eu/ctr-search/trial/2009-011795-29/FI

33. **Claghorn 1983**
Claghorn J, Gershon S, Goldstein BJ. Zimeldine tolerability in comparison to amitriptyline and placebo. Acta Psychiatr Scand Suppl. 1983;308:104-14.

34. **Claghorn 1995 (MIR 003-002 (FDA)**
Claghorn JL, Lesem MD. A double-blind placebo-controlled study of Org 3770 in depressed outpatients. J Affect Disord. 1995 Jun 8;34(3):165-71.

35. **Claghorn 1996**
Claghorn JL, Earl CQ, Walczak DD, Stoner KA, Wong LF, Kanter D, Houser VP. Fluvoxamine maleate in the treatment of depression: a single-center, double-blind, placebo-controlled comparison with imipramine in outpatients. J Clin Psychopharmacol. 1996 Apr;16(2):113-20.

36. **Clayton 2003 (Study 050)**
Clayton AH, Zajecka J, Ferguson JM, Filipiak-Reisner JK, Brown MT, Schwartz GE. Lack of sexual dysfunction with the selective noradrenaline reuptake inhibitor reboxetine during treatment for major depressive disorder. Int Clin Psychopharmacol. 2003;18(3):151-6. https://www.iqwig.de/de/projekte-ergebnisse/studieninformationen-zu-reboxetin.3304.html

37. **Clayton 2006a (WELL AK130926)**
Clayton AH, Croft H, Horrigan JP, et al. Bupropion XL compared with escitalopram: effects on sexual functioning and antidepressant efficacy in two randomized, double-blind, placebo controlled studies. J Clin Psychiatry 2006; 67: 736–46. https://www.gsk-clinicalstudyregister.com/files2/20466.pdf

38. **Clayton 2006b (WELL AK130927)**

Clayton AH, Croft H, Horrigan JP, et al. Bupropion XL compared with escitalopram: effects on sexual functioning and antidepressant efficacy in two randomized, double-blind, placebo controlled studies. J Clin Psychiatry 2006; 67: 736–46. https://www.gsk-clinicalstudyregister.com/files2/20467.pdf

39. **Clayton 2013 (NCT01121484)**
Clayton AH, Kornstein SG, Dunlop BW, Focht K, Musgnung J, Ramey T, Bao W, Ninan PT. Efficacy and safety of desvenlafaxine 50 mg/d in a randomized, placebo-controlled study of perimenopausal and postmenopausal women with major depressive disorder. J Clin Psychiatry. 2013 Oct;74(10):1010-7. https://clinicaltrials.gov/ct2/show/NCT01121484

40. **Clayton 2015 (NCT01432457)**
Clayton AH, Tourian KA, Focht K, Hwang E, Cheng RF, Thase ME. Desvenlafaxine 50 and 100 mg/d versus placebo for the treatment of major depressive disorder: a phase 4, randomized controlled trial. J Clin Psychiatry. 2015 May;76(5):562-9.
https://clinicaltrials.gov/show/NCT01432457

41. **CN104-002 (FDA)**
http://digitalcommons.ohsu.edu/fdadrug/23/
https://digitalcollections.ohsu.edu/concern/etds/3484zh30d?locale=en

42. **CN104-045 (FDA)**
http://digitalcommons.ohsu.edu/fdadrug/23/
https://digitalcollections.ohsu.edu/concern/etds/3484zh30d?locale=en

43. **CN104-054 (FDA)**
http://digitalcommons.ohsu.edu/fdadrug/23/
https://digitalcollections.ohsu.edu/concern/etds/3484zh30d?locale=en

44. **Cohn 1985a**
Cohn JB, Wilcox C. A comparison of fluoxetine, imipramine, and placebo in patients with major depressive disorder. J Clin Psychiatry. 1985 Mar;46(3 Pt 2):26-31.

45. **Cohn 1996 (FDA CN104-006-2)**
Cohn CK, Robinson DS, Roberts DL, Schwiderski UE, O'Brien K, Ieni JR. Responders to antidepressant drug treatment: a study comparing nefazodone, imipramine, and placebo in patients with major depression. J Clin Psychiatry. 1996;57 Suppl 2:15-8.
http://digitalcommons.ohsu.edu/fdadrug/23/
https://digitalcollections.ohsu.edu/concern/etds/3484zh30d?locale=en

46. **Coleman 1999 (AK1A4002)**
Coleman CC, Cunningham LA, Foster VJ, et al. Sexual dysfunction associated with the treatment of depression: a placebo-controlled comparison of bupropion sustained release and sertraline treatment. Ann Clin Psychiatry 1999; 11: 205–15.
http://www.gsk-clinicalstudyregister.com/study/AK1A4002#rs

47. **Coleman 2001 (AK1A4007)**
Coleman CC, King BR, Bolden-Watson C, et al. A placebo-controlled comparison of the eff ects on sexual functioning of bupropion sustained release and fluoxetine. Clin Ther 2001; 23: 1040–58.
http://www.gsk-clinicalstudyregister.com/study/AK1A4007#rs

48. **Corrigan 2000**
Corrigan MH, Denahan AQ, Wright CE, Ragual RJ, Evans DL. Comparison of pramipexole, fluoxetine, and placebo in patients with major depression. Depress Anxiety 2000;11(2):58-65.

49. **Croft 1999 (AK1A4001)**

Croft H, Settle E Jr, Houser T, Batey SR, Donahue RMJ, Ascher JA. A placebo-controlled comparison of the antidepressant efficacy and effects on sexual functioning of sustained-release bupropion and sertraline. Clin Ther 1999; 21: 643–58.
http://www.gsk-clinicalstudyregister.com/study/AK1A4001#rs

50. **Croft 2014 (NCT01473394)**
Croft HA, Pomara N, Gommoll C, Chen D, Nunez R, Mathews M. Efficacy and safety of vilazodone in major depressive disorder: a randomized, double-blind, placebo-controlled trial. J Clin Psychiatry. 2014 Nov;75(11):e1291-8.
https://clinicaltrials.gov/ct2/show/NCT01473394

51. **Cunningham 1994 (VEN 600A-302 FDA)**
Cunningham LA, Borison RL, Carman JS, Chouinard G, Crowder JE, Diamond BI, Fischer DE, Hearst E. A comparison of venlafaxine, trazodone, and placebo in major depression. J Clin Psychopharmacol. 1994 Apr;14(2):99-106.
http://digitalcommons.ohsu.edu/fdadrug/24/
https://digitalcollections.ohsu.edu/concern/etds/pk02c992m?locale=en

52. **Cunningham 1997 (VEN XR 208 - FDA)**
Cunningham LA. Once-daily venlafaxine extended release (XR) and venlafaxine immediate release (IR) in outpatients with major depression. Venlafaxine XR 208 Study Group. Ann Clin Psychiatry. 1997 Sep;9(3):157-64.
http://digitalcommons.ohsu.edu/fdadrug/25/
https://digitalcollections.ohsu.edu/concern/etds/z029p503q?locale=en

53. **Cutler 2009**
Cutler AJ, Montgomery SA, Feifel D, Lazarus A, Aström M, Brecher M. Extended release quetiapine fumarate monotherapy in major depressive disorder: a placebo- and duloxetine-controlled study. J Clin Psychiatry. 2009 Apr;70(4):526-39.

54. **D'Amico 1990 (FDA 030-A2-0007)**
D'Amico MF, Roberts DL, Robinson DS, Schwiderski UE, Copp J. Placebo-controlled dose-ranging trial designs in phase II development of nefazodone. Psychopharmacol Bull. 1990;26(1):147-50.
http://digitalcommons.ohsu.edu/fdadrug/23/
https://digitalcollections.ohsu.edu/concern/etds/3484zh30d?locale=en

55. **Davidson 2002 (HDTSG, NCT00005013)**
Hypericum Depression Trial Study Group. Effect of Hypericum perforatum (St John's wort) in major depressive disorder: a randomized controlled trial. JAMA. 2002 Apr 10;287(14):1807-14.
https://www.clinicaltrials.gov/ct2/show/NCT00005013

56. **DeMartinis 2007 (Study 306, NCT00072774)**
DeMartinis NA, Yeung PP, Entsuah R, Manley AL. A double-blind, placebo-controlled study of the efficacy and safety of desvenlafaxine succinate in the treatment of major depressive disorder. J Clin Psychiatry. 2007 May;68(5):677-88.
http://www.accessdata.fda.gov/drugsatfda_docs/nda/2008/021992s000TOC.cfm
https://clinicaltrials.gov/ct2/show/NCT00072774

57. **Detke 2002a (HMBH - Study Group A)**
Detke MJ, Lu Y, Goldstein DJ, Hayes JR, Demitrack MA. Duloxetine, 60 mg once daily, for major depressive disorder: a randomized double-blind placebo-controlled trial. J Clin Psychiatry. 2002 Apr;63(4):308-15.

58. **Detke 2002b (HMBH - Study Group B)**
Detke MJ, Lu Y, Goldstein D, McNamara R, Demitrack M. Duloxetine 60 mg once daily dosing versus placebo in the acute treatment of major depression. J Psychiatr Res 2002;36:383-90.

59. **Detke 2004 (HMAY Study Group A)**

Detke MJ, Wiltse CG, Mallinckrodt CH, McNamara RK, Demitrack MA, Bitter I. Duloxetine in the acute and long-term treatment of major depressive disorder: a placebo- and paroxetine-controlled trial. Eur Neuropsychopharmacol 2004; 14: 457–70.
http://www.lillytrials.com/results/Cymbalta.pdf

60. **Dimidjian 2006**
Dimidjian S, Hollon SD, Dobson KS, Schmaling KB, Kohlenberg RJ, Addis ME, Gallop R, McGlinchey JB, Markley DK, Gollan JK, Atkins DC, Dunner DL, Jacobson NS. Randomized trial of behavioral activation, cognitive therapy, and antidepressant medication in the acute treatment of adults with major depression. J Consult Clin Psychol. 2006 Aug;74(4):658-70.

61. **Dominguez 1985**
Dominguez RA, Goldstein BJ, Jacobson AF, Steinbook RM. A double-blind placebo-controlled study of fluvoxamine and imipramine in depression. J Clin Psychiatry. 1985;46(3):84-7.

62. **Doogan 1994**
Doogan D, Langdon C. Double-blind, placebo-controlled comparison of sertraline and dothiepin in treatment of major depression in general practice. Int Clin Psychopharmacol 1994;9:95-100.

63. **Dube 2010 (NCT00420004)**
Dubé S, Dellva MA, Jones M, Kielbasa W, Padich R, Saha A, Rao P. A study of the effects of LY2216684, a selective norepinephrine reuptake inhibitor, in the treatment of major depression. J Psychiatr Res. 2010 Apr;44(6):356-63
https://clinicaltrials.gov/ct2/show/NCT00420004

64. **Dunbar 1993a (Claghorn1992, Rickels1989, Rickels1992, PAR 02-001 - FDA)**
Claghorn JL. The safety and efficacy of paroxetine compared with placebo in a double-blind trial of depressed outpatients. J Clin Psychiatry. 1992 Feb;53 Suppl:33-5.
http://digitalcommons.ohsu.edu/fdadrug/26/
https://digitalcollections.ohsu.edu/concern/etds/9s161642r?locale=en

65. **Dunbar 1993b (Claghorn1992, PAR 02-002 - FDA)**
Dunbar GC, Claghorn JL, Kiev A, Rickels K, Smith WT. A comparison of paroxetine and placebo in depressed outpatients. Acta Psychiatr Scand. 1993;87(5):302-5
http://digitalcommons.ohsu.edu/fdadrug/26/
https://digitalcollections.ohsu.edu/concern/etds/9s161642r?locale=en

66. **Dunbar 1993c (Smith1992, PAR 02-003 - FDA)**
Smith WT, Glaudin V. A placebo-controlled trial of paroxetine in the treatment of major depression. J Clin Psychiatry. 1992;53 Suppl:36-9.
http://digitalcommons.ohsu.edu/fdadrug/26/
https://digitalcollections.ohsu.edu/concern/etds/9s161642r?locale=en

67. **Dunbar 1993d (Kiev1992, PAR 02-004 - FDA)**
Kiev A. A double-blind, placebo-controlled study of paroxetine in depressed outpatients. J Clin Psychiatry. 1992;53 Suppl:27-9.
http://digitalcommons.ohsu.edu/fdadrug/26/
https://digitalcollections.ohsu.edu/concern/etds/9s161642r?locale=en

68. **Dunlop 2011 (NCT00824291)**
Dunlop BW, Reddy S, Yang L, Lubaczewski S, Focht K, Guico-Pabia CJ. Symptomatic and functional improvement in employed depressed patients: a double-blind clinical trial of desvenlafaxine versus placebo. J Clin Psychopharmacol. 2011 Oct;31(5):569-76.
https://clinicaltrials.gov/ct2/show/NCT00824291

69. **Dunner 1992 (PAR 29060.09)**

Dunner DL, Dunbar GC. Optimal dose regimen for paroxetine. J Clin Psychiatry. 1992;53 Suppl:21-6.
http://www.gsk-clinicalstudyregister.com/study/29060/009#rs

70. **Edwards 1989 (MD/PAR/009 PAR-276)**
Edwards JG, Goldie A. Placebo-controlled trial of paroxetine in depressive illness. Human Psychopharmacology 1993;8:203-209
http://www.gsk-clinicalstudyregister.com/study/29060/276?search=compound&compound=paroxetine#rs

71. **Fabre 1979**
Fabre LF, McLendon DM, Gainey A. Trazodone efficacy in depression: a double-blind comparison with imipramine and placebo in day-hospital type patients. Current Therapeutic research 1979;25:827-834

72. **Fabre 1995 (SER 103 FDA)**
Fabre L, Abuzzahab F, Amin M, Claghorn J, Mendels J, Dubé S, Small JG. Sertraline in major depression: double-blind comparison with placebo. Biol Psychiatry 1995;38:592-602.
http://digitalcommons.ohsu.edu/fdadrug/27/
https://digitalcollections.ohsu.edu/concern/etds/nk322d61z?locale=en

73. **Fabre 1996**
Fabre L, Birkhimer L, Zaborny B, Wong L, Kapik B. Fluvoxamine vs imipramine and placebo: a double-blind comparison in depressed patients. Int Clin Psychopharmacol 1996;11:119-27.

74. **Fava 1998**
Fava M, Amsterdam JD, Deltito JA. A double-blind study of paroxetine, fluoxetine, and placebo in outpatients with major depression. Ann Clin Psychiatry 1998; 10: 145–50.

75. **Fava 2005**
Fava M, Alpert J, Nierenberg AA, Mischoulon D, Otto MW, Zajecka J, Murck H, Rosenbaum JF. A Double-blind, randomized trial of St John's wort, fluoxetine, and placebo in major depressive disorder. J Clin Psychopharmacol. 2005 Oct;25(5):441-7.

76. **Feiger2009 (Study 320)**
Feiger AD, Tourian KA, Rosas GR, Padmanabhan SK. A placebo-controlled study evaluating the efficacy and safety of flexible-dose desvenlafaxine treatment in outpatients with major depressive disorder. CNS Spectr. 2009;14(1):41-50.
http://www.accessdata.fda.gov/drugsatfda_docs/nda/2008/021992s000TOC.cfm

77. **Feighner 1979**
Feighner JP, Brauzer B, Gelenberg AJ, Gomez E, Kiev A, Kurland ML, Weiss BL. A placebo-controlled multicenter trial of Limbitrol versus its components (amitriptyline and chlordiazepoxide) in the symptomatic treatment of depressive illness. Psychopharmacology (Berl). 1979 Mar 22;61(2):217-25.

78. **Feighner 1980**
Feighner JP. Trazodone, a triazolopyridine derivative, in primary depressive disorder. J Clin Psychiatry. 1980;41(7):250-5.

79. **Feighner 1984**
Feighner JP, Meredith CH, Stern WC, Hendrickson G, Miller LL. A double-blind study of bupropion and placebo in depression. Am J Psychiatry. 1984;141(4):525-9.

80. **Feighner 1989a**
Feighner JP, Boyer WF, Merideth CH, Hendrickson GG. A double-blind comparison of fluoxetine, imipramine and placebo in outpatients with major depression. Int Clin Psychopharmacol. 1989 Apr;4(2):127-34.

81. **Feighner 1989b**

Feighner JP, Boyer WF, Meredith CH, Hendrickson GG. A placebo-controlled inpatient comparison of fluvoxamine maleate and imipramine in major depression. Int Clin Psychopharmacol. 1989 Jul;4(3):239-44.

82. **Feighner 1993a (Feighner 1989c, PAR 03 001 - FDA)**
Feighner JP, Boyer WF. Paroxetine in the treatment of depression: a comparison with imipramine and placebo. Acta Psychiatr Scand Suppl. 1989;350:125-9.
`http://digitalcommons.ohsu.edu/fdadrug/26/`
`https://digitalcollections.ohsu.edu/concern/etds/9s161642r?locale=en`

83. **Feighner 1993b (Cohn1990, Cohn1992, PAR 03 002 - FDA)**
Cohn JB, Wilcox CS. Paroxetine in major depression: a double-blind trial with imipramine and placebo. J Clin Psychiatry. 1992 Feb;53 Suppl:52-6.
`http://digitalcommons.ohsu.edu/fdadrug/26/`
`https://digitalcollections.ohsu.edu/concern/etds/9s161642r?locale=en`

84. **Feighner 1993c (PAR 03 003 - FDA)**
Dunbar GC, Cohn JB, Fabre LF, Feighner JP, Fieve RR, Mendels J, Shrivastava RK.
A comparison of paroxetine, imipramine and placebo in depressed out-patients. Br J Psychiatry. 1991 Sep;159:394-8
`http://digitalcommons.ohsu.edu/fdadrug/26/`
`https://digitalcollections.ohsu.edu/concern/etds/9s161642r?locale=en`

85. **Feighner 1993d (Shrivastava 1992, PAR 03 004 - FDA)**
Shrivastava RK, Shrivastava SH, Overweg N, Blumhardt CL. A double-blind comparison of paroxetine, imipramine and placebo in major depression. J Clin Psychiatry 1992;53 Suppl:48-51.
`http://digitalcommons.ohsu.edu/fdadrug/26/`
`https://digitalcollections.ohsu.edu/concern/etds/9s161642r?locale=en`

86. **Feighner 1993e (PAR 03 005 - FDA)**
Feighner JP. Cohn JB. Fabre LF Jr. Fieve RR. Mendels J. Shrivastava RK. Dunbar GC. A study comparing paroxetine placebo and imipramine in depressed patients. Journal of Affective Disorders. 28(2):71-79, 1993.
`http://digitalcommons.ohsu.edu/fdadrug/26/`
`https://digitalcollections.ohsu.edu/concern/etds/9s161642r?locale=en`

87. **Feighner 1993f (Fabre 1992, PAR 03 006 - FDA)**
Fabre LF. A 6-week, double-blind trial of paroxetine, imipramine, and placebo in depressed outpatients. J Clin Psychiatry. 1992;53 Suppl:40-3.
`http://digitalcommons.ohsu.edu/fdadrug/26/`
`https://digitalcollections.ohsu.edu/concern/etds/9s161642r?locale=en`

88. **Feighner 1998**
Feighner J, Targum SD, Bennett ME, Roberts DL, Kensler TT, D'Amico MF, Hardy SA. A double-blind, placebo-controlled trial of nefazodone in the treatment of patients hospitalized for major depression. J Clin Psychiatry. 1998 May;59(5):246-53.

89. **Feighner 1999 (Study 91206 FDA)**
Feighner JP1, Overø K. Multicenter, placebo-controlled, fixed-dose study of citalopram in moderate-to-severe depression. J Clin Psychiatry. 1999 Dec;60(12):824-30.

90. **Fontaine 1994 (FDA 03A0A-003)**
Fontaine R, Ontiveros A, Elie R, Kensler TT, Roberts DL, Kaplita S, Ecker JA, Faludi G. A double-blind comparison of nefazodone, imipramine, and placebo in major depression. J Clin Psychiatry. 1994;55(6):234-41.
`http://digitalcommons.ohsu.edu/fdadrug/23/`
`https://digitalcollections.ohsu.edu/concern/etds/3484zh30d?locale=en`

91. **Frank 2004**

Frank MG, Hendricks SE, Burke WJ, Johnson DR. Clinical response augments NK cell activity independent of treatment modality: a randomized double-blind placebo controlled antidepressant trial. Psychol Med. 2004 Apr;34(3):491-8.

92. **Gastpar 2006**
Gastpar M, Singer A, Zeller K. Comparative efficacy and safety of a once-daily dosage of hypericum extract STW3-VI and citalopram in patients with moderate depression: a double-blind, randomised, multicentre, placebo-controlled study. Pharmacopsychiatry. 2006 Mar;39(2):66-75.

93. **Gelenberg 1990**
Gelenberg AJ, Wojcik JD, Falk WE, Spring B, Brotman AW, Galvin-Nadeau M. Clovoxamine in the treatment of depressed outpatients: a double-blind, parallel-group comparison against amitriptyline and placebo. Compr Psychiatry. 1990 Jul-Aug;31(4):307-14.

94. **Georgotas 1982**
Georgotas A, Krakowski M, Gershon S. Controlled trial of zimelidine, a 5-HT reuptake inhibitor, for treatment of depression. Am J Psychiatry. 1982 Aug;139(8):1057-8.

95. **Gerner 1980**
Gerner R, Estabrook W, Steuer J, Jarvik L. Treatment of geriatric depression with trazodone, imipramine, and placebo: a double-blind study. J Clin Psychiatry. 1980 Jun;41(6):216-20.

96. **Golden 2002a (29060/448)**
Golden RN, Nemeroff CB, McSorley P, Pitts CD, Dubé EM. Efficacy and tolerability of controlled-release and immediate-release paroxetine in the treatment of depression. J Clin Psychiatry. 2002 Jul;63(7):577-84.
`http://www.gsk-clinicalstudyregister.com/study/29060/448#rs`

97. **Golden 2002b (29060/449)**
Golden RN, Nemeroff CB, McSorley P, Pitts CD, Dubé EM. Efficacy and tolerability of controlled-release and immediate-release paroxetine in the treatment of depression. J Clin Psychiatry. 2002 Jul;63(7):577-84.
`http://www.gsk-clinicalstudyregister.com/study/29060/449#rs`

98. **Goldstein 2002 (HMAQ - Study Group A)**
Goldstein DJ, Mallinckrodt C, Lu Y, Demitrack MA. Duloxetine in the treatment of major depressive disorder: a double-blind clinical trial. J Clin Psychiatry. 2002; 63: 225–31.
`http://www.lillytrials.com/results/Cymbalta.pdf`

99. **Goldstein 2004a (HMAT - Study Group A, ID#4091)**
`http://www.lillytrials.com/results/Cymbalta.pdf`

100. **Goldstein 2004b (HMAT - Study Group B, ID#4091)**
Goldstein DJ, Lu Y, Detke MJ, Wiltse C, Mallinckrodt C, Demitrack MA. Duloxetine in the treatment of depression: a double-blind placebo-controlled comparison with paroxetine. J Clin Psychopharmacol. 2004; 24: 389–99.

101. **Gommoll 2014 (LVM-MD-02, NCT00969150)**
Gommoll CP, Greenberg WM, Chen C. A randomized, double-blind, placebo-controlled study of flexible doses of levomilnacipran ER (40–120 mg/day) in patients with major depressive disorder. Journal of Drug Assessment. 2014;3(1):10-19

102. **Gorman 2002 (SCT-MD-02)**
Gorman JM, Korotzer A, Su G. Efficacy comparison of escitalopram and citalopram in the treatment of major depressive disorder: pooled analysis of placebo-controlled trials. CNS Spectr. 2002 Apr;7(4 Suppl 1):40-4.
`www.ncbi.nlm.nih.gov/pubmedhealth/PMH0016624/bin/app17c_et6.pdf`

103. **Griebel 2012 (Study DF15878, NCT00358631)**

Griebel G, Beeské S, Stahl SM. The vasopressin V(1b) receptor antagonist SSR149415 in the treatment of major depressive and generalized anxiety disorders: results from 4 randomized, double-blind, placebo-controlled studies. J Clin Psychiatry. 2012 Nov;73(11):1403-11.
https://clinicaltrials.gov/ct2/show/NCT00358631

104. **Griebel 2012b (Study DF15879, NCT00361491)**
Griebel G, Beeské S, Stahl SM. The vasopressin V(1b) receptor antagonist SSR149415 in the treatment of major depressive and generalized anxiety disorders: results from 4 randomized, double-blind, placebo-controlled studies. J Clin Psychiatry. 2012 Nov;73(11):1403-11.
https://clinicaltrials.gov/ct2/show/NCT00361491

105. **GSK14**
http://www.gsk-clinicalstudyregister.com/study/14#rs

106. **Guelfi 1995 (VEN 600A-206 FDA)**
Guelfi JD, White C, Hackett D, Guichoux JY, Magni G. Effectiveness of venlafaxine in patients hospitalized for major depression and melancholia. J Clin Psychiatry. 1995 Oct;56(10):450-8.
http://digitalcommons.ohsu.edu/fdadrug/24/
https://digitalcollections.ohsu.edu/concern/etds/pk02c992m?locale=en

107. **Halikas 1995 (MIR 003-023 - FDA)**
Halikas JA. Org 3770 (Mirtazapine) versus Trazodone: A Placebo Controlled Trial in Depressed Elderly Patients. Human Psychopharmacology 1995;10:S125-S133.
http://digitalcommons.ohsu.edu/fdadrug/20/
https://digitalcollections.ohsu.edu/concern/etds/p8418n49j?locale=en

108. **Heiligenstein 1994**
Heiligenstein J, Faries D, Rush A, Andersen J, Pande A, Roffwarg H, Dunner D, Gillin J, James S, Lahmeyer H. Latency to rapid eye movement sleep as a predictor of treatment response to fluoxetine and placebo in nonpsychotic depressed outpatients. Psychiatry Res. 1994;52:327-39.

109. **Henigsberg 2012 (305, NCT00735709)**
Henigsberg N, Mahableshwarkar AR, Jacobsen P, Chen Y, Thase ME. A randomized, double-blind, placebo-controlled 8-week trial of the efficacy and tolerability of multiple doses of Lu AA21004 in adults with major depressive disorder. J Clin Psychiatry 2012;73(7):953-9.
https://clinicaltrials.gov/ct2/show/NCT00735709

110. **Heun 2013 (lSRCTł57507360)**
Heun R, Ahokas A, Boyer P, Giménez-Montesinos N, Pontes-Soares F, Olivier V; Agomelatine Study Group. The efficacy of agomelatine in elderly patients with recurrent major depressive disorder: a placebo-controlled study. J Clin Psychiatry. 2013 Jun;74(6):587-94.

111. **Hewett 2009 (WXL101497)**
Hewett K, Chrzanowski W, Schmitz M, Savela A, Milanova V, Gee M, Krishen A, Millen L, Leary MO, Modell J. Eight-week, placebo-controlled, double-blind comparison of the antidepressant efficacy and tolerability of bupropion XR and venlafaxine XR. J Psychopharmacol. 2009;23(5):531-8.
http://www.gsk-clinicalstudyregister.com/study/101497#rs

112. **Hewett 2010a (AK130940, NCT00093288)**
Hewett K, Chrzanowski W, Schmitz M, Savela A, Milanova V, Gee M, Krishen A, Millen L, Leary MO, Modell J. Eight-week, placebo-controlled, double-blind comparison of the antidepressant efficacy and tolerability of bupropion XR and venlafaxine XR. J Psychopharmacol. 2009;23(5):531-8.
http://www.gsk-clinicalstudyregister.com/study/AK130940#ps
https://clinicaltrials.gov/ct2/show/NCT00093288

113.**Hewett 2010b (AK130939)**
Hewett K, Gee MD, Krishen A, Wunderlich HP, Le Clus A, Evoniuk G, Modell JG. Double-blind, placebo-controlled comparison of the antidepressant efficacy and tolerability of bupropion XR and venlafaxine XR. J Psychopharmacol. 2010;24(8):1209-16.
`http://www.gsk-clinicalstudyregister.com/study/AK130939#rs`
114.**Hicks 1988**
Hicks F, Robins E, Murphy GE. Comparison of adinazolam, amitriptyline, and placebo in the treatment of melancholic depression. Psychiatry Res. 1988 Feb;23(2):221-7.
115.**Higuchi 2014 (NCT01441440)**
Higuchi T, Kamijima K, Nakagome K, Itamura R, Asami Y, Kuribayashi K, Imaeda T. A randomized, double-blinded, placebo-controlled study to evaluate the efficacy and safety of venlafaxine extended release and a long-term extension study for patients with major depressive disorder in Japan. Int Clin Psychopharmacol. 2016 Jan;31(1):8-19.
`https://clinicaltrials.gov/ct2/show/NCT01441440`
116.**Higuchi 2009**
Higuchi T, Murasaki M, Kamijima K. Clinical evaluation of duloxetine in the treatment of major depressive disorder. Jpn J Clin Psychopharmacol 2009;12:1613-1634.
117.**Higuchi 2011 (PCR112810, NCT00866294)**
Higuchi T, Hong JP, Jung HY, Watanabe Y, Kunitomi T, Kamijima K. Paroxetine controlled-release formulation in the treatment of major depressive disorder: a randomized, double-blind, placebo-controlled study in Japan and Korea. Psychiatry Clin Neurosci 2011;65:655-63.
`http://www.gsk-clinicalstudyregister.com/study/112810#ps`
`https://clinicaltrials.gov/ct2/show/NCT00866294`
118.**Hirayasu 2011a**
Hirayasu Y. A dose-response study of escitalopram in patients with major depressive disorder: a placebo-controlled, double-blind study. Jpn J Clin Psychopharmacol 2011;14:871-882.
119.**Hirayasu 2011b**
Hirayasu Y. A dose-response and non-inferiority study evaluating the efficacy and safety of escitalopram in patients with major depressive disorder. Jpn J Clin Psychopharmacol 2011;14:883-899.
120.**Hormazabal 1985**
Hormazabal L, Omer LM, Ismail S. Cianopramine and amitriptyline in the treatment of depressed patients–a placebo-controlled study. Psychopharmacology (Berl) 1985;86(1-2):205-8.
121.**Hunter 2010 (Study 1)**
Hunter AM, Muthén BO, Cook IA, Leuchter AF. Antidepressant response trajectories and quantitative electroencephalography (QEEG) biomarkers in major depressive disorder. J Psychiatr Res. 2010 Jan;44(2):90-8.
122.**Hunter 2010 (Study 2)**
Hunter AM, Muthén BO, Cook IA, Leuchter AF. Antidepressant response trajectories and quantitative electroencephalography (QEEG) biomarkers in major depressive disorder. J Psychiatr Res. 2010 Jan;44(2):90-8.
123.**Hunter 2010 (Study 3)**
Hunter AM, Muthén BO, Cook IA, Leuchter AF. Antidepressant response trajectories and quantitative electroencephalography (QEEG) biomarkers in major depressive disorder. J Psychiatr Res. 2010 Jan;44(2):90-8.
124.**Itil 1983**
Itil TM, Shrivastava RK, Mukherjee S, Coleman BS, Michael ST. A double-blind placebo-controlled study of fluvoxamine and imipramine in out-patients with primary depression. Br J Clin Pharmacol. 1983;15(Suppl 3):433S-438S.
125.**Iwata 2013 (NCT00798707)**

Iwata N1, Tourian KA, Hwang E, Mele L, Vialet C. Efficacy and safety of desvenlafaxine 25 and 50mg/day in a randomized, placebo-controlled study of depressed outpatients. J Psychiatr Pract. 2013 Jan;19(1):5-14.
https://clinicaltrials.gov/ct2/show/NCT00798707

126. **Jacobsen 2015 (316, NCT01163266)**
Jacobsen PL, Mahableshwarkar AR, Serenko M, Chan S, Trivedi MH. A randomized, double-blind, placebo-controlled study of the efficacy and safety of vortioxetine 10 mg and 20 mg in adults with major depressive disorder. J Clin Psychiatry 2015;76(5):575-82.
https://clinicaltrials.gov/ct2/show/NCT01163266

127. **Jain2013 (303, NCT00672958)**
Jain R, Mahableshwarkar AR, Jacobsen PL, Chen Y, Thase ME. A randomized, double-blind, placebo-controlled 6-wk trial of the efficacy and tolerability of 5 mg vortioxetine in adults with major depressive disorder. Int J Neuropsychopharmacol. 2013;16(2):313-21.
https://clinicaltrials.gov/ct2/show/NCT00672958

128. **Jefferson 2000 (29060/785)**
Jefferson J, Griest J. A double-blind, placebo controlled, fixed-dosage study comparing the efficacy and tolerability of paroxetine CR and citalopram to placebo in the treatment of Major Depressive Disorder with anxiety. American College of Neuropsychopharmacology, 39th Annual Meeting 12/11/2000 San Juan, Puerto Rico.
http://www.gsk-clinicalstudyregister.com/study/29060/785#csr

129. **Jefferson 2006**
Jefferson JW, Rush AJ, Nelson JC, VanMeter SA, Krishen A, Hampton KD, Wightman DS, Modell JG. Extended-release bupropion for patients with major depressive disorder presenting with symptoms of reduced energy, pleasure, and interest: findings from a randomized, double-blind, placebo-controlled study. J Clin Psychiatry. 2006 Jun;67(6):865-73.

130. **Kane 1983**
Kane JM, Cole K, Sarantakos S, Howard A, Borenstein M. Safety and efficacy of bupropion in elderly patients: preliminary observations. J Clin Psychiatry. 1983 May;44(5 Pt 2):134-6.

131. **Kasper 2005a (Study 99024)**
Kasper S, de Swart H, Friis Andersen H. Escitalopram in the treatment of depressed elderly patients. Am J Geriatr Psychiatry. 2005 Oct;13(10):884-91.

132. **Kasper 2012 (NCT00807248)**
Kasper S, Ebert B, Larsen K, Tonnoir B. Combining escitalopram with gaboxadol provides no additional benefit in the treatment of patients with severe major depressive disorder. Int J Neuropsychopharmacol. 2012 Jul;15(6):715-25.
https://clinicaltrials.gov/ct2/show/NCT00807248

133. **Katona 2012 (12541A, NCT00811252)**
Katona C, Hansen T, Olsen CK. A randomized, double-blind, placebo-controlled, duloxetine-referenced, fixed-dose study comparing the efficacy and safety of Lu AA21004 in elderly patients with major depressive disorder. Int Clin Psychopharmacol 2012;27(4):215-23.
https://clinicaltrials.gov/ct2/show/NCT00811252

134. **Katz 1993a**
Katz RJ, Lott M, Landau P, Waldmeier P. A clinical test of noradrenergic involvement in the therapeutic mode of action of an experimental antidepressant. Biol Psychiatry. 1993 Feb 15;33(4):261-6.

135. **Katz 1993b**
Katz RJ, Lott M, Landau P, Waldmeier P. A clinical test of noradrenergic involvement in the therapeutic mode of action of an experimental antidepressant. Biol Psychiatry. 1993 Feb 15;33(4):261-6.

136. **Katz 2004**

Katz MM, Tekell JL, Bowden CL, Brannan S, Houston JP, Berman N, Frazer A. Onset and early behavioral effects of pharmacologically different antidepressants and placebo in depression. Neuropsychopharmacology. 2004;29(3):566-79.

137. **Kellams 1979**
Kellams JJ, Klapper M, Small J. Trazodone, a new antidepressant: efficacy and safety in endogenous depression. J Clin Psychiatry. 1979;40(9):390-5.

138. **Keller 2006a (Study059, NCT00035009)**
Keller M, Montgomery S, Ball W, Morrison M, Snavely D, Liu G, Hargreaves R, Hietala J, Lines C, Beebe K, Reines S. Lack of efficacy of the substance p (neurokinin1 receptor) antagonist aprepitant in the treatment of major depressive disorder. Biol Psychiatry. 2006 Feb 1;59(3):216-23.
`https://clinicaltrials.gov/ct2/show/NCT00035009`

139. **Keller 2006b (Study061, NCT00035295)**
Keller M, Montgomery S, Ball W, Morrison M, Snavely D, Liu G, Hargreaves R, Hietala J, Lines C, Beebe K, Reines S. Lack of efficacy of the substance p (neurokinin1 receptor) antagonist aprepitant in the treatment of major depressive disorder. Biol Psychiatry. 2006 Feb 1;59(3):216-23.
`https://clinicaltrials.gov/ct2/show/NCT00035295?term=NCT00035295&rank=1`

140. **Keller 2006c (Study062, NCT00048607)**
Keller M, Montgomery S, Ball W, Morrison M, Snavely D, Liu G, Hargreaves R, Hietala J, Lines C, Beebe K, Reines S. Lack of efficacy of the substance p (neurokinin1 receptor) antagonist aprepitant in the treatment of major depressive disorder. Biol Psychiatry. 2006 Feb 1;59(3):216-23.
`https://clinicaltrials.gov/ct2/show/NCT00048607?term=NCT00048607&rank=1`

141. **Kennedy 2006 (CL3-20098-043)**
Kennedy, SH, Emsley, R Placebo-controlled trial of agomelatine in the treatment of major depressive disorder. Eur Neuropsychopharm 2006; 16: 93-100.
`http://www.ema.europa.eu/docs/en_GB/document_library/EPAR_-_Public_assessment_report/human/000916/WC500038315.pdf`

142. **Kennedy 2014 (EudraCT 2009-011238-84, CL3-20098-069)**
Kennedy SH, Avedisova A, Giménez-Montesinos N, Belaïdi C, de Bodinat C; Agomelatine Study Group. A placebo-controlled study of three agomelatine dose regimens (10 mg, 25 mg, 25-50 mg) in patients with major depressive disorder. Eur Neuropsychopharmacol. 2014 Apr;24(4):553-63.
`https://www.clinicaltrialsregister.eu/ctr-search/search?query=2009-011238-84`
`http://www.ema.europa.eu/docs/en_GB/document_library/EPAR_-_Public_assessment_report/human/000916/WC500038315.pdf`

143. **Khan 1998**
Khan A, Upton GV, Rudolph RL, Entsuah R, Leventer SM. The use of venlafaxine in the treatment of major depression and major depression associated with anxiety: a dose-response study. Venlafaxine Investigator Study Group. J Clin Psychopharmacol. 1998 Feb;18(1):19-25.

144. **Khan 2011 (CLDA-07-DP-02, NCT00683592)**
Khan A, Cutler AJ, Kajdasz DK, Gallipoli S, Athanasiou M, Robinson DS, Whalen H, Reed CR. A randomized, double-blind, placebo-controlled, 8-week study of vilazodone, a serotonergic agent for the treatment of major depressive disorder. J Clin Psychiatry. 2011;72(4):441-7.
`https://clinicaltrials.gov/ct2/show/NCT00683592`

145. **Kinoshita 2009**
Kinoshita T. A double-blind, placebo controlled study of a new antidepressant, mirtazapine, in depressed patients. Jpn J Clin Psychopharmacol 2009;12:289-306.

146.**Kornstein2010 (NCT00369343)**
Kornstein SG, Jiang Q, Reddy S, Musgnung JJ, Guico-Pabia CJ. Short-term efficacy and safety of desvenlafaxine in a randomized, placebo-controlled study of perimenopausal and postmenopausal women with major depressive disorder. J Clin Psychiatry 2010;71(8):1088-96.
147.**Koshino 2013 (NCT01138007)**
Koshino Y, Bahk W, Sakai H, Kobayashi T. Efficacy and safety of bupropion sustained-release formulation for the treatment of major depressive disorder: a multi-center, randomized, double-blind, placebo-controlled study in Asian patients. Neuropsychiatr Dis Treat. 2013;9:1273-80.
https://clinicaltrials.gov/ct2/show/NCT01138007
148.**Kramer 1998**
Kramer MS, Cutler N, Feighner J, Shrivastava R, Carman J, Sramek JJ, Reines SA, Liu G, Snavely D, Wyatt-Knowles E, Hale JJ, Mills SG, MacCoss M, Swain CJ, Harrison T, Hill RG, Hefti F, Scolnick EM, Cascieri MA, Chicchi GG, Sadowski S, Williams AR, Hewson L, Smith D, Carlson EJ, Hargreaves RJ, Rupniak NM. Distinct mechanism for antidepressant activity by blockade of central substance P receptors. Science. 1998 Sep 11;281(5383):1640-5.
149.**Kusalic 1993**
Kusalic M1, Engelsmann F, Bradwejn J. Thyroid functioning during treatment for depression. J Psychiatry Neurosci. 1993;18(5):260-3.
150.**Lam 1995**
Lam RW, Gorman CP, Michalon M, Steiner M, Levitt AJ, Corral MR, Watson GD, Morehouse RL, Tam W, Joffe RT. Multicenter, placebo-controlled study of fluoxetine in seasonal affective disorder. Am J Psychiatry. 1995;152(12):1765-70.
151.**Langlois 1985**
Langlois R, Cournoyer G, de Montigny C, Caillé G. High incidence of multisystemic reactions to zimeldine. Eur J Clin Pharmacol. 1985;28(1):67-71.
152.**Lapierre 1987**
Lapierre YD, Browne M, Horn E, Oyewumi LK, Sarantidis D, Roberts N, Badoe K, Tessier P. Treatment of major affective disorder with fluvoxamine. J Clin Psychiatry. 1987 Feb;48(2):65-8.
153.**Larsen 1989**
Larsen JK, Holm P, Høyer E, Mejlhede A, Mikkelsen PL, Olesen A, Schaumburg E. Moclobemide and clomipramine in reactive depression. A placebo-controlled randomized clinical trial. Acta Psychiatr Scand. 1989 Jun;79(6):530-6.
154.**Learned 2012a Study1 (EUCTR2005-003401-87, SND103285, NCT00448058)**
Learned S, Graff O, Roychowdhury S, Moate R, Krishnan KR, Archer G, Modell JG, Alexander R, Zamuner S, Lavergne A, Evoniuk G, Ratti E. Efficacy, safety, and tolerability of a triple reuptake inhibitor GSK372475 in the treatment of patients with major depressive disorder: two randomized, placebo- and active-controlled clinical trials. J Psychopharmacol. 2012;26:653-62.
https://www.clinicaltrialsregister.eu/ctr-search/search?query=2005-003401-87 https://clinicaltrials.gov/ct2/show/NCT00448058
155.**Learned 2012b Study2 (NCT00420641)**
Learned S, Graff O, Roychowdhury S, Moate R, Krishnan KR, Archer G, Modell JG, Alexander R, Zamuner S, Lavergne A, Evoniuk G, Ratti E. Efficacy, safety, and tolerability of a triple reuptake inhibitor GSK372475 in the treatment of patients with major depressive disorder: two randomized, placebo- and active-controlled clinical trials. J Psychopharmacol. 2012 May;26(5):653-62.
https://clinicaltrials.gov/ct2/show/NCT00420641
156.**Lecrubier 1997**

Lecrubier Y, Bourin M, Moon CA, Schifano F, Blanchard C, Danjou P, Hackett D. Efficacy of venlafaxine in depressive illness in general practice. Acta Psychiatr Scand. 1997;95(6):485-93.

157. **Lepola 2003 (ESC 99003)**
Lepola UM, Loft H, Reines EH .Escitalopram (10-20 mg/day) is effective and well tolerated in a placebo-controlled study in depression inprimary care. Int Clin Psychopharmacol. 2003 Jul;18(4):211-7.

158. **Lieberman 2008a (Study 309, EUCTR2004-000562-13, 3151A1-309-EU, NCT00090649)**
Lieberman DZ, Montgomery SA, Tourian KA, Brisard C, Rosas G, Padmanabhan K, Germain JM, Pitrosky B. A pooled analysis of two placebo-controlled trials of desvenlafaxine in major depressive disorder. Int Clin Psychopharmacol. 2008 Jul;23(4):188-97
`https://www.clinicaltrialsregister.eu/ctr-search/search?query=2004-000562-13` `https://clinicaltrials.gov/ct2/show/NCT00090649`

159. **Lieberman 2008b (Study 317)**
Lieberman DZ, Montgomery SA, Tourian KA, Brisard C, Rosas G, Padmanabhan K, Germain JM, Pitrosky B. A pooled analysis of two placebo-controlled trials of desvenlafaxine in major depressive disorder. Int Clin Psychopharmacol. 2008 Jul;23(4):188-97

160. **Liebowitz2007 (NCT00063206)**
Liebowitz MR, Yeung PP, Entsuah R. A randomized, double-blind, placebo-controlled trial of desvenlafaxine succinate in adult outpatients with major depressive disorder. J Clin Psychiatry. 2007 Nov;68(11):1663-72.

161. **Liebowitz 2008 (Study 332, NCT00277823)**
Liebowitz MR, Manley AL, Padmanabhan SK, Ganguly R, Tummala R, Tourian KA. Efficacy, safety, and tolerability of desvenlafaxine 50 mg/day and 100 mg/day in outpatients with major depressive disorder. Curr Med Res Opin. 2008;24(7):1877-90. `https://clinicaltrials.gov/ct2/show/NCT00277823`

162. **Liebowitz 2013 (NCT00863798)**
Liebowitz MR1, Tourian KA, Hwang E, Mele L; Study 3362 Investigators. A double-blind, randomized, placebo-controlled study assessing the efficacy and tolerability of desvenlafaxine 10 and 50 mg/day in adult outpatients with major depressive disorder. BMC Psychiatry. 2013 Mar 22;13:94.
`https://www.clinicaltrials.gov/ct2/show/NCT00863798`

163. **Lineberry 1990 (WELL 84A)**
Lineberry CG, Johnston JA, Raymond RN, Samara B, Feighner JP, Harto NE, Granacher RP Jr, Weisler RH, Carman JS, Boyer WF. A fixed-dose (300 mg) efficacy study of bupropion and placebo in depressed outpatients. J Clin Psychiatry. 1990;51(5):194-9.
`http://www.gsk-clinicalstudyregister.com/study/Well%2084A#rs`

164. **Loo 2002 (CL2-014)**
Lôo H, Hale A, D'haenen H. Determination of the dose of agomelatine, a melatoninergic agonist and selective 5-HT(2C) antagonist, in the treatment of major depressive disorder: a placebo-controlled dose range study. Int Clin Psychopharmacol. 2002 Sep;17(5):239-47.

165. **Lopez-Rodriguez 2004**
Rodriguez JL, Lopez Butron MA, Vargas Terrez BE, Villamil Salcedo V. Estudio doble ciego con antidepressivo, psicoterapia breve y placebo en pacientes con depression leve a moderada. Salud Mental 2004;27:53-61.

166. **Lydiard 1989**
Lydiard RB, Laird LK, Morton WA Jr, Steele TE, Kellner C, Laraia MT, Ballenger JC. Fluvoxamine, imipramine, and placebo in the treatment of depressed outpatients: effects on depression. Psychopharmacol Bull. 1989;25(1):68-70.

167. **Lydiard 1997**
Lydiard RB1, Stahl SM, Hertzman M, Harrison WM. A double-blind, placebo-controlled study comparing the effects of sertraline versus amitriptyline in the treatment of major depression. J Clin Psychiatry. 1997 Nov;58(11):484-91.

168.**M/2020/0046 (Study 046)**
https://www.iqwig.de/de/projekte-ergebnisse/studieninformationen-zu-reboxetin.3304.html
169.**M/2020/0047 (Study 047)**
https://www.iqwig.de/de/projekte-ergebnisse/studieninformationen-zu-reboxetin.3304.html
170.**Mahableshwarkar 2013 (304, NCT00672620)**
Mahableshwarkar AR, Jacobsen PL, Chen Y. A randomized, double-blind trial of 2.5?mg and 5?mg vortioxetine (Lu AA21004) versus placebo for 8 weeks in adults with major depressive disorder. Curr Med Res Opin. 2013;29(3):217-26.
https://clinicaltrials.gov/ct2/show/NCT00672620
171.**Mahableshwarkar 2015a (315, NCT01153009)**
Mahableshwarkar AR, Jacobsen PL, Chen Y, Serenko M, Trivedi MH. A randomized, double-blind, duloxetine-referenced study comparing efficacy and tolerability of 2 fixed doses of vortioxetine in the acute treatment of adults with MDD. Psychopharmacology (Berl). 2015;232(12):2061-70.
https://clinicaltrials.gov/ct2/show/NCT01153009
172.**Mahableshwarkar 2015b (317, NCT01179516)**
Mahableshwarkar AR, Jacobsen PL, Serenko M, Chen Y, Trivedi MH. A randomized, double-blind, placebo-controlled study of the efficacy and safety of 2 doses of vortioxetine in adults with major depressive disorder. J Clin Psychiatry 2015;76(5):583-91.
https://clinicaltrials.gov/ct2/show/NCT01179516
173.**Mahableshwarkar 2015c (NCT01564862)**
Mahableshwarkar AR, Zajecka J, Jacobson W, Chen Y, Keefe RS. A Randomized, Placebo-Controlled, Active-Reference, Double-Blind, Flexible-Dose Study of the Efficacy of Vortioxetine on Cognitive Function in Major Depressive Disorder. Neuropsychopharmacology. 2015;40(8):2025-37.
https://clinicaltrials.gov/ct2/show/NCT01564862
174.**Mann 1981**
Mann JJ, Georgotas A, Newton R, Gershon S. A controlled study of trazodone, imipramine, and placebo in outpatients with endogenous depression. J Clin Psychopharmacol. 1981;1(2):75-80.
175.**Mao 2015 (NCT01098318)**
Mao JJ, Xie SX, Zee J, Soeller I, Li QS, Rockwell K, Amsterdam JD. Rhodiola rosea versus sertraline for major depressive disorder: A randomized placebo-controlled trial. Phytomedicine. 2015 Mar 15;22(3):394-9.
https://clinicaltrials.gov/ct2/show/NCT01098318
176.**March 1990**
March JS, Kobak KA, Jefferson JW, Mazza J, Greist JH. A double-blind, placebo-controlled trial of fluvoxamine versus imipramine in outpatients with major depression. J Clin Psychiatry. 1990 May;51(5):200-2.
177.**Mathews 2015 (NCT01473381)**
Mathews M, Gommoll C, Chen D, Nunez R, Khan A. Efficacy and safety of vilazodone 20 and 40?mg in major depressive disorder: a randomized, double-blind, placebo-controlled trial. Int Clin Psychopharmacol. 2015;30(2):67-74.
https://clinicaltrials.gov/ct2/show/NCT01473381
178.**McGrath 2000**
McGrath PJ, Stewart JW, Janal MN, Petkova E, Quitkin FM, Klein DF. A placebo-controlled study of fluoxetine versus imipramine in the acute treatment of atypical depression. Am J Psychiatry. 2000 Mar;157(3):344-50.
179.**McIntyre 2014 (NCT01422213)**

McIntyre RS, Lophaven S, Olsen CK. A randomized, double-blind, placebo-controlled study of vortioxetine on cognitive function in depressed adults. Int J Neuropsychopharmacol. 2014;17(10):1557-67.
https://clinicaltrials.gov/ct2/show/NCT01422213

180. **MDUK/26090/III/83/007**
https://www.gsk-clinicalstudyregister.com/study/26090/007#rs

181. **Mendels 1995 (FDA 03A0A-004B)**
Mendels J, Reimherr F, Marcus RN, Roberts DL, Francis RJ, Anton SF. A double-blind, placebo-controlled trial of two dose ranges of nefazodone in the treatment of depressed outpatients. J Clin Psychiatry. 1995;56 Suppl 6:30-6.
http://digitalcommons.ohsu.edu/fdadrug/23/
https://digitalcollections.ohsu.edu/concern/etds/3484zh30d?locale=en

182. **Mendels 1999 (Study 85A - FDA)**
Mendels J, Kiev A, Fabre LF. Double blind comparison of citalopram and placebo in depressed outpatients with melancholia. Depress Anxiety. 1999;9(2):54-60.
http://www.accessdata.fda.gov/drugsatfda_docs/nda/98/020822a.cfm

183. **Miller 1989 (MDUK/29060/III/82/006, PAR-274, PAR UK 06 - FDA)**
Miller SM, Naylor GJ, Murtagh M, Winslow G. A double-blind comparison of paroxetine and placebo in the treatment of depressed patients in a psychiatric outpatient clinic. Acta Psychiatr Scand Suppl. 1989;350:143-4
http://digitalcommons.ohsu.edu/fdadrug/26/
https://digitalcollections.ohsu.edu/concern/etds/9s161642r?locale=en

184. **MIR 003-003 (FDA)**
http://digitalcommons.ohsu.edu/fdadrug/20/
https://digitalcollections.ohsu.edu/concern/etds/p8418n49j?locale=en

185. **MIR 003-020 (FDA)**
http://digitalcommons.ohsu.edu/fdadrug/20/
https://digitalcollections.ohsu.edu/concern/etds/p8418n49j?locale=en

186. **MIR 003-021 (FDA)**
http://digitalcommons.ohsu.edu/fdadrug/20/
https://digitalcollections.ohsu.edu/concern/etds/p8418n49j?locale=en

187. **MIR 84062 (FDA)**
http://digitalcommons.ohsu.edu/fdadrug/20/
https://digitalcollections.ohsu.edu/concern/etds/p8418n49j?locale=en

188. **Mischoulon 2014 (NCT00101452)**
A Double-Blind, Randomized, Placebo-Controlled Clinical Trial of S-Adenosyl-l-Methionine (SAMe) Versus Escitalopram in Major Depressive Disorder. The Journal of Clinical Psychiatry. 2014;75(4):370-76.
https://clinicaltrials.gov/ct2/show/NCT00101452

189. **Montgomery 1992 (Study 89303 FDA)**
http://www.accessdata.fda.gov/drugsatfda_docs/nda/98/020822a.cfm

190. **Montgomery 2013 (F02695LP202, EudraCT2006-002404-34)**
Montgomery SA, Mansuy L, Ruth A, Bose A, Li H, Li D. Efficacy and safety of levomilnacipran sustained release in moderate to severe major depressive disorder: a randomized, double-blind, placebo-controlled, proof-of-concept study. J Clin Psychiatry 2013;74(4):363-9.
https://www.clinicaltrialsregister.eu/ctr-search/search?query=2006-002404-34

191. **Moreno 2005**

Moreno RA, Teng CT, Almeida KM, Tavares Junior H. Hypericum perforatum versus fluoxetine in the treatment of mild to moderate depression: a randomized double-blind trial in a Brazilian sample. Rev Bras Psiquiatr. 2006 Mar;28(1):29-32.

192.**Moscovitch 2004**
Moscovitch A, Blashko CA, Eagles JM, Darcourt G, Thompson C, Kasper S, Lane RM; International Collaborative Group on Sertraline in the Treatment of Outpatients with Seasonal Affective Disorders. A placebo-controlled study of sertraline in the treatment of outpatients with seasonal affective disorder. Psychopharmacology (Berl). 2004 Feb;171(4):390-7.

193.**Mundt 2012 (NCT00406952)**
Mundt JC, Vogel AP, Feltner DE, Lenderking WR. Vocal acoustic biomarkers of depression severity and treatment response. Biol Psychiatry. 2012 Oct 1;72(7):580-7.
`https://clinicaltrials.gov/ct2/show/NCT00406952`

194.**MY-1008/BRL-029060/2/CPMS-076**
`https://www.gsk-clinicalstudyregister.com/study/29060/076#rs`

195.**MY-1042/BRL-029060/CPMS-251**
`http://www.gsk-clinicalstudyregister.com/study/29060/251?search=compound&compound=paroxetine#rs`

196.**MY-1043/BRL-029060/115**
`http://www.gsk-clinicalstudyregister.com/study/29060/115#rs`

197.**MY-1045/BRL-029060/1 (PAR 128)**
`http://www.gsk-clinicalstudyregister.com/study/29060/128#rs`

198.**Mynors-Wallis 1995**
Mynors-Wallis LM, Gath DH, Lloyd-Thomas AR, Tomlinson D. Randomised controlled trial comparing problem solving treatment with amitriptyline and placebo for major depression in primary care. BMJ 1995 Feb 18;310(6977):441-5.

199.**NCT00822744 (EudraCT Number2008-001718-26)**
`https://clinicaltrials.gov/ct2/show/NCT00822744`
`https://www.clinicaltrialsregister.eu/ctr-search/trial/2008-001718-26/SK`

200.**NCT01020799**
`https://clinicaltrials.gov/ct2/show/NCT01020799`

201.**NCT01145755**
`https://clinicaltrials.gov/ct2/show/NCT01145755`

202.**NCT01254305**
`https://clinicaltrials.gov/ct2/show/NCT01254305`

203.**NCT01255787 (EUCTR2010-022257-41, JapicCTI-101344, CTRI/2011/08/001963)**
`https://clinicaltrials.gov/ct2/show/NCT01255787`
`https://www.clinicaltrialsregister.eu/ctr-search/search?query=2010-022257-41`

204.**NCT01355081 (japicCTI-111492, U1111-1120-9277)**
`https://clinicaltrials.gov/ct2/show/NCT01355081`

205.**NCT01808612**
`https://clinicaltrials.gov/ct2/show/NCT01808612`

206.**Nemeroff 2007**
Nemeroff CB, Thase ME; EPIC 014 Study Group. A double-blind, placebo-controlled comparison of venlafaxine and fluoxetine treatment in depressed outpatients. J Psychiatry Res 2007;41: 351–59.

207.**Nierenberg 2007 (F1J-MC-HMCR, NCT00073411, Pigott 2007)**

Nierenberg AA, Greist JH, Mallinckrodt CH, et al. Duloxetine versus escitalopram and placebo in the treatment of patients with major depressive disorder: onset of antidepressant action, a non-inferiority study. Curr Med Res Opin 2007; 23: 401–16.
http://www.lillytrials.com/results/Cymbalta.pdf https://clinicaltrials.gov/ct2/show/NCT00073411

208. **Ninan 2003 (poster SCT-MD-26)**
www.ncbi.nlm.nih.gov/pubmedhealth/PMH0016624/bin/app17c_et6.pdf

209. **NKD20006 (NCT00048204)**
http://www.gsk-clinicalstudyregister.com/study/NKD20006#ps https://clinicaltrials.gov/ct2/show/NCT00048204

210. **Norton 1984**
Norton KR, Sireling LI, Bhat AV, Rao B, Paykel ES. A double-blind comparison of fluvoxamine, imipramine and placebo in depressed patients. J Affect Disord. 1984;7:297-308.

211. **Oakes 2012a (NCT00536471)**
Oakes TM, Myers AL, Marangell LB, Ahl J, Prakash A, Thase ME, Kornstein SG. Assessment of depressive symptoms and functional outcomes in patients with major depressive disorder treated with duloxetine versus placebo: primary outcomes from two trials conducted under the same protocol. Hum Psychopharmacol. 2012 Jan;27(1):47-56.
https://www.clinicaltrials.gov/ct2/show/NCT00536471

212. **Oakes 2012b (NCT00536471)**
Oakes TM, Myers AL, Marangell LB, Ahl J, Prakash A, Thase ME, Kornstein SG. Assessment of depressive symptoms and functional outcomes in patients with major depressive disorder treated with duloxetine versus placebo: primary outcomes from two trials conducted under the same protocol. Hum Psychopharmacol. 2012 Jan;27(1):47-56.
https://www.clinicaltrials.gov/ct2/show/NCT00536471

213. **Olie 1997**
Olie J, Gunn K, Katz E. A double-blind placebo-controlled multicentre study of sertraline in the acute and continuation treatment of major depression. Eur Psychiatry. 1997;12(1):34-41.

214. **Olie 2007 (CL3-20098-042)**
Olie, JP, Kasper, S Efficacy of agomelatine, a MT1/MT2 receptor agonist with 5-HT2C antagonistic properties, in major depressive disorder. Int J Neuropsychoph 2007; 10: 661-73.
http://www.ema.europa.eu/docs/en_GB/document_library/EPAR_-_Public_assessment_report/human/000916/WC500038315.pdf

215. **PAR 01 001 (GSK & FDA)**
http://digitalcommons.ohsu.edu/fdadrug/26/
https://digitalcollections.ohsu.edu/concern/etds/9s161642r?locale=en

216. **PAR 279 MDUK**
http://www.gsk-clinicalstudyregister.com/study/29060/012_3?search=compound&compound=paroxetine#rs

217. **Paykel 1988**
Paykel ES, Hollyman JA, Freeling P, Sedgwick P. Predictors of therapeutic benefit from amitriptyline in mild depression: a general practice placebo-controlled trial. J Affect Disord. 1988 Jan-Feb;14(1):83-95.

218. **Perahia 2006 (HMAY - Study Group B)**
Perahia DG, Wang F, Mallinckrodt CH, Walker DJ, Detke MJ. Duloxetine in the treatment of major depressive disorder: a placebo- and paroxetine-controlled trial. Eur Psychiatry 2006; 21: 367–78.
http://www.lillytrials.com/results/Cymbalta.pdf

219. **Pomara 2013**

Croft HA, Pomara N, Gommoll C, Chen D, Nunez R, Mathews M. Efficacy and Safety of Vilazodone in Major Depressive Disorder: A Randomized, Double-blind, Placebo-controlled Trial. J Clin Psychiatry. 2014 Nov;75(11):e1291-8.

220. **PZ/109**
Hieronymus F, Emilsson JF, Nilsson S, Eriksson E. Consistent superiority of selective serotonin reuptake inhibitors over placebo in reducing depressed mood in patients with major depression. Mol Psychiatry. 2016 Apr;21(4):523-30.

221. **PZ/111**
Hieronymus F, Emilsson JF, Nilsson S, Eriksson E. Consistent superiority of selective serotonin reuptake inhibitors over placebo in reducing depressed mood in patients with major depression. Mol Psychiatry. 2016 Apr;21(4):523-30.

222. **Raft 1981**
Raft D, Davidson J, Wasik J, Mattox A. Relationship between response to phenelzine and MAO inhibition in a clinical trial of phenelzine, amitriptyline and placebo. Neuropsychobiology. 1981;7(3):122-6.

223. **Rapaport 2003 (PAR487)**
Rapaport MH, Schneider LS, Dunner DL, Davies JT, Pitts CD. Efficacy of controlled-release paroxetine in the treatment of late-life depression. J Clin Psychiatry 2003;64(9):1065-74.
http://www.gsk-clinicalstudyregister.com/study/29060/487#rs

224. **Rapaport 2009 (BRL-29060/874, NCT00067444)**
Rapaport MH, Lydiard RB, Pitts CD, Schaefer D, Bartolic EI, Iyengar M, Carfagno M, Lipschitz A. Low doses of controlled-release paroxetine in the treatment of late-life depression: a randomized, placebo-controlled trial. J Clin Psychiatry. 2009 Jan;70(1):46-57.
https://www.clinicaltrials.gov/ct2/show/NCT00067444

225. **Raskin 2007 (HMBV, NCT00062673)**
Raskin J, Wiltse CG, Siegal A, Sheikh J, Xu J, Dinkel JJ, Rotz BT, Mohs RC. Efficacy of duloxetine on cognition, depression, and pain in elderly patients with major depressive disorder: an 8-week, double-blind, placebo-controlled trial. Am J Psychiatry 2007;164(6):900-9.
http://www.lillytrials.com/results/Cymbalta.pdf https://clinicaltrials.gov/ct2/show/NCT00062673

226. **Ravindran 1995**
Ravindran AV, Teehan MD, Bakish D, Yatham L, O'Reilly R, Fernando ML, Manchanda R, Charbonneau Y, Buttars J. The impact of sertraline, desipramine, and placebo on psychomotor functioning in depression. Human Psychopharmacology. 1995; 10(4):273–281.

227. **Reimherr 1990 (SER 104 - FDA)**
Reimherr FW, Chouinard G, Cohn CK, Cole JO, Itil TM, LaPierre YD, Masco HL, Mendels J. Antidepressant efficacy of sertraline: a double-blind, placebo- and amitriptyline-controlled, multicenter comparison study in outpatients with major depression. J Clin Psychiatry. 1990 Dec;51 Suppl B:18-27.
http://digitalcommons.ohsu.edu/fdadrug/27/
https://digitalcollections.ohsu.edu/concern/etds/nk322d61z?locale=en

228. **Reimherr 1998 (WELL203)**
Reimherr FW, Cunningham LA, Batey SR, Johnston JA, Ascher JA. A multicenter evaluation of the efficacy and safety of 150 and 300 mg/d sustained-
release bupropion tabletsversus placebo in depressed outpatients. Clin Ther. 1998 May-Jun;20(3):505-16.
http://digitalcommons.ohsu.edu/fdadrug/21/
https://digitalcollections.ohsu.edu/concern/etds/8k71nh40n?locale=en

229. **Rickels 1982**
Rickels K, Case WG. Trazodone in depressed outpatients. Am J Psychiatry. 1982 Jun;139(6):803-6.

230. **Rickels 1985**
Rickels K, Feighner JP, Smith WT. Alprazolam, amitriptyline, doxepin, and placebo in the treatment of depression. Arch Gen Psychiatry. 1985 Feb;42(2):134-41.
231. **Rickels 1994 (FDA CN104-005)**
Rickels K, Schweizer E, Clary C, Fox I, Weise C. Nefazodone and imipramine in major depression: a placebo-controlled trial. Br J Psychiatry. 1994;164(6):802-5.
`http://digitalcommons.ohsu.edu/fdadrug/23/`
`https://digitalcollections.ohsu.edu/concern/etds/3484zh30d?locale=en`
232. **Rickels 1995 (FDA CN104-006-1)**
Rickels K, Robinson DS, Schweizer E, Marcus RN, Roberts DL. Nefazodone: aspects of efficacy. J Clin Psychiatry. 1995;56 Suppl 6:43-6.
`http://digitalcommons.ohsu.edu/fdadrug/23/`
`https://digitalcollections.ohsu.edu/concern/etds/3484zh30d?locale=en`
233. **Rickels 2009 (GNSC-04-DP-02, NCT00285376)**
Rickels K, Athanasiou M, Robinson DS, Gibertini M, Whalen H, Reed CR. Evidence for efficacy and tolerability of vilazodone in the treatment of major depressive disorder: a randomized, double-blind, placebo-controlled trial. J Clin Psychiatry. 2009 Mar;70(3):326-33.
`https://clinicaltrials.gov/ct2/show/NCT00285376`
234. **Robinson 2014 (NCT00406848)**
Robinson M, Oakes TM, Raskin J, Liu P, Shoemaker S, Nelson JC. Acute and long-term treatment of late-life major depressive disorder: duloxetine versus placebo. Am J Geriatr Psychiatry. 2014 Jan;22(1):34-45.
`https://clinicaltrials.gov/ct2/show/NCT00406848`
235. **Roffman 1982**
Roffman M, Gould EE. A Double-blind comparative study of oxaprotiline with amitriptyline and placebo in moderate depression. Current Therapeutic Research 1982;32:2247-256.
236. **Roose 2004 (CIT-MD-03)**
Roose SP, Sackeim HA, Krishnan KR, Pollock BG, Alexopoulos G, Lavretsky H, Katz IR, Hakkarainen H; Old-Old Depression Study Group. Antidepressant pharmacotherapy in the treatment of depression in the very old: a randomized, placebo-controlled trial. Am J Psychiatry. 2004 Nov;161(11):2050-9.
237. **Roth 1990**
Roth D, Mattes J, Sheehan KH, Sheehan DV. A double-blind comparison of fluvoxamine, desipramine and placebo in outpatients with depression. Progress in Neuro-Psychopharmacology and Biological Psychiatry. 1990;14(6):929–939.
238. **Rowan 1980**
Rowan P, Paykel ES, Parker PR, West E. Comparative effects of phenelzine and amitriptyline: a placebo controlled trial. Neuropharmacology. 1980 Dec;19(12):1223-5.
239. **Rudolph 1998 (VEN 600A-203 (FDA)**
Rudolph RL, Fabre LF, Feighner JP, Rickels K, Entsuah R, Derivan AT. A randomized, placebo-controlled, dose-response trial of venlafaxine hydrochloride in the treatment of major depression. J Clin Psychiatry. 1998 Mar;59(3):116-22.
`http://digitalcommons.ohsu.edu/fdadrug/24/`
`https://digitalcollections.ohsu.edu/concern/etds/pk02c992m?locale=en`
240. **Rudolph 1999**
Rudolph RL, Feiger AD. A double-blind, randomized, placebo-controlled trial of once-daily venlafaxine extended release (xr) and fluoxetine for the treatment of depression. J Affect Disord 1999; 56: 171–81.
241. **Sambunaris 2014 (LVM-MD-03, NCT01034462)**

Sambunaris A, Bose A, Gommoll CP, Chen C, Greenberg WM, Sheehan DV. A phase III, double-blind, placebo-controlled, flexible-dose study of levomilnacipran extended-release in patients with major depressive disorder. J Clin Psychopharmacol. 2014 Feb;34(1):47-56.
https://clinicaltrials.gov/ct2/show/NCT01034462

242. **Schatzberg 2006a**
Schatzberg A1, Roose S. A double-blind, placebo-controlled study of venlafaxine and fluoxetine in geriatric outpatients with major depression. Am J Geriatr Psychiatry. 2006 Apr;14(4):361-70.

243. **Schneider 2003**
Schneider LS, Nelson JC, Clary CM, Newhouse P, Krishnan KR, Shiovitz T, Weihs K; Sertraline Elderly Depression Study Group. An 8-week multicenter, parallel-group, double-blind, placebo-controlled study of sertraline in elderly outpatients with major depression. Am J Psychiatry. 2003 Jul;160(7):1277-85.

244. **Schweizer 1994 (VEN 600A-301 FDA)**
Schweizer E, Feighner J, Mandos LA, Rickels K. Comparison of venlafaxine and imipramine in the acute treatment of major depression in outpatients. J Clin Psychiatry. 1994 Mar;55(3):104-8.
http://digitalcommons.ohsu.edu/fdadrug/24/
https://digitalcollections.ohsu.edu/concern/etds/pk02c992m?locale=en

245. **SCT-MD-49 (NCT00668525)**
https://clinicaltrials.gov/ct2/show/NCT00668525

246. **Septien-Velez2007 (Study 308)**
Septien-Velez L, Pitrosky B, Padmanabhan SK, Germain JM, Tourian KA. A randomized, double-blind, placebo-controlled trial of desvenlafaxine succinate in the treatment of major depressive disorder. Int Clin Psychopharmacol. 2007;22(6):338-47
http://www.accessdata.fda.gov/drugsatfda_docs/nda/2008/021992s000TOC.cfm

247. **SER 101 (FDA)**
http://digitalcommons.ohsu.edu/fdadrug/27/
https://digitalcollections.ohsu.edu/concern/etds/nk322d61z?locale=en

248. **SER 310 (FDA)**
http://digitalcommons.ohsu.edu/fdadrug/27/
https://digitalcollections.ohsu.edu/concern/etds/nk322d61z?locale=en

249. **SER 315 (FDA)**
http://digitalcommons.ohsu.edu/fdadrug/27/
https://digitalcollections.ohsu.edu/concern/etds/nk322d61z?locale=en

250. **Settle 1999 (WELL212)**
Settle EC, Stahl SM, Batey SR, Johnston JA, Ascher JA. Safety profile of sustained-release bupropion in depression: results of three clinical trials. Clin Ther. 1999;21(3):454-63.
http://digitalcommons.ohsu.edu/fdadrug/21/
https://digitalcollections.ohsu.edu/concern/etds/8k71nh40n?locale=en

251. **Sheehan 2009a**
Sheehan DV, Nemeroff CB, Thase ME, Entsuah R; EPIC 016 Study Group. Placebo-controlled inpatient comparison of venlafaxine and fluoxetine for the treatment of major depression with melancholic features. Int Clin Psychopharmacol. 2009 Mar;24(2):61-86.

252. **Sheehan 2009b (NCT00775203)**
Sheehan DV, Croft HA, Gossen ER, Levitt RJ, Brullé C, Bouchard S, Rozova A. Extended-release Trazodone in Major Depressive Disorder: A Randomized, Double-blind, Placebo-controlled Study. Psychiatry (Edgmont). 2009;6(5):20-33.

https://clinicaltrials.gov/ct2/show/NCT00775203

253. **Shipley 1981**
Shipley JE, Kupfer DJ, Spiker DG, Shaw DH, Coble PA, Neil JF, Cofsky J. Neuropsychological assessment and EEG sleep in affective disorders. Biol Psychiatry. 1981 Oct;16(10):907-18.

254. **Silverstone 1999**
Silverstone PH, Ravindran A. Once-daily venlafaxine extended release (xr) compared with fluoxetine in outpatients with depression and anxiety. Venlafaxine xr 360 study group. J Clin Psychiatry 1999; 60: 22–28.

255. **Smith 1990 (MIR 003-024 FDA)**
Smith WT, Glaudin V, Panagides J, Gilvary E. Mirtazapine vs Amitriptyline vs Placebo in the treatment of major depressive disorder. Psychopharmacologi Bulltin 1990;26:191-195.
http://digitalcommons.ohsu.edu/fdadrug/20/
https://digitalcollections.ohsu.edu/concern/etds/p8418n49j?locale=en

256. **Sramek 1995**
Sramek JJ, Kashkin K, Jasinsky O, Kardatzke D, Kennedy S, Cutler NR. Placebo-controlled study of ABT-200 versus fluoxetine in the treatment of major depressive disorder. Depression 1995;3:199-203.

257. **Stahl 2000**
Stahl SM. Placebo-controlled comparison of the selective serotonin reuptake inhibitors citalopram and sertraline. Biol Psychiatry. 2000 Nov 1;48(9):894-901.

258. **Stahl 2010 (CAGO178A2302)**
Stahl, SM, Fava, M, Trivedi, MH, Caputo, A, Shah, A, Post, A Agomelatine in the treatment of major depressive disorder: an 8-week, multicenter, randomized, placebo-controlled trial. J Clin Psychiat 2010; 71: 616-26.
https://clinicaltrials.gov/ct2/show/NCT00411242

259. **Stark 1985 (Study 27 - FDA)**
Stark P, Hardison CD. A review of multicenter controlled studies of fluoxetine vs. imipramine and placebo in outpatients with major depressive disorder. J Clin Psychiatry 1985;46:53-8.
http://digitalcommons.ohsu.edu/fdadrug/22/
https://digitalcollections.ohsu.edu/concern/etds/5h73pw36j?locale=en

260. **Stratas 1984**
Stratas NE. A double-blind study of the efficacy and safety of dothiepin hydrochloride in the treatment of major depressive disorder. J Clin Psychiatry. 1984 Nov;45(11):466-9.

261. **Studie009 (CTN009-FCE20124)**
https://www.iqwig.de/de/projekte-ergebnisse/studieninformationen-zu-reboxetin.3304.html

262. **Study 015**
https://www.iqwig.de/de/projekte-ergebnisse/studieninformationen-zu-reboxetin.3304.html

263. **Study 032a (CTN032-FCE20124)**
https://www.iqwig.de/de/projekte-ergebnisse/studieninformationen-zu-reboxetin.3304.html

264. **Study 045**
https://www.iqwig.de/de/projekte-ergebnisse/studieninformationen-zu-reboxetin.3304.html

265. **Study 049**
https://www.iqwig.de/de/projekte-ergebnisse/studieninformationen-zu-reboxetin.3304.html

266.**Study 19 (FDA) (Fabre 1985)**
Fabre, LF, Crismon, L. Efficacy of fluoxetine in outpatients with major depression. Current Therapeutic Research. 1985;37(1):115-23.
http://digitalcommons.ohsu.edu/fdadrug/22/
https://digitalcollections.ohsu.edu/concern/etds/5h73pw36j?locale=en
267.**Study 205 (FDA) (WELL 205)**
http://digitalcommons.ohsu.edu/fdadrug/21/
https://digitalcollections.ohsu.edu/concern/etds/8k71nh40n?locale=en
268.**Study 25 (FDA) (Rickels 1986)**
Rickels K, Amsterdam JD, Avallone MF. Fluoxetine in major depression: A controlled study. Current Therapeutic Research. 1986;39(4):559-63.
http://digitalcommons.ohsu.edu/fdadrug/22/
https://digitalcollections.ohsu.edu/concern/etds/5h73pw36j?locale=en
269.**Study 62a (FDA) - MILD depression (Dunlop 1990)**
Dunlop SR, Dornseif BE, Wernicke JR, Potvin JH. Pattern analysis shows beneficial effect of fluoxetine treatment in mild depression. Psychopharmacology bulletin. 1990;26(2):173-80.
http://digitalcommons.ohsu.edu/fdadrug/22/

https://digitalcollections.ohsu.edu/concern/etds/5h73pw36j?locale=en
270.**Study 62b (FDA) - MODERATE depression**
http://digitalcommons.ohsu.edu/fdadrug/22/

https://digitalcollections.ohsu.edu/concern/etds/5h73pw36j?locale=en
271.**Study 89306 (FDA)**
http://www.accessdata.fda.gov/drugsatfda_docs/nda/98/020822a_medr_P2.pdf
272.**Study F1J-MC-HMAQ - Study Group B (starting page in the pdf document: 147)**
http://www.lillytrials.com/results/Cymbalta.pdf
273.**Thase 1997 (VEN XR 209 FDA)**
Thase ME. Efficacy and tolerability of once-daily venlafaxine extended release (XR) in outpatients with major depression. J Clin Psychiatry. 1997;58(9):393-8.
http://digitalcommons.ohsu.edu/fdadrug/25/
https://digitalcollections.ohsu.edu/concern/etds/z029p503q?locale=en
274.**Thomson 1982**
Thomson J, Rankin H, Ashcroft GW, Yates CM, McQueen JK, Cummings SW. The treatment of depression in general practice: a comparison of L-tryptophan, amitriptyline, and a combination of L-tryptophan and amitriptyline with placebo. Psychol Med. 1982 Nov;12(4):741-51.
275.**Tollefson 1995**
Tollefson GD, Bosomworth JC, Heiligenstein JH, Potvin JH, Holman S. A double-blind, placebo-controlled clinical trial of fluoxetine in geriatric patients with major depression. The Fluoxetine Collaborative Study Group. Int Psychogeriatr. 1995 Spring;7(1):89-104.
276.**Tomarken 2004**
Tomarken AJ, Dichter GS, Freid C, Addington S, Shelton RC. Assessing the effects of bupropion SR on mood dimensions of depression. J Affect Disord. 2004 Mar;78(3):235-41.
277.**Tourian 2009 (NCT00384033)**

Tourian KA, Padmanabhan SK, Groark J. Desvenlafaxine 50 and 100 mg/d in the Treatment of Major Depressive Disorder: An 8-Week, Phase III, Multicenter, Randomized, Double-Blind, Placebo-Controlled, Parallel Group Trial and a Post Hoc Pooled Analysis of Three Studies. Clinical Therapeutics 2009;31:1405-1423.
https://clinicaltrials.gov/show/NCT00384033

278. **Trivedi 2004 (29060/810)**
Trivedi MH, Pigotti TA, Perera P, Dillingham KE, Carfagno ML, Pitts CD. Effectiveness of low doses of paroxetine controlled release in the treatment of major depressive disorder. J Clin Psychiatry. 2004;65(10):1356-64.
http://www.gsk-clinicalstudyregister.com/study/29060/810?search=study&#rs

279. **Van de Merwe 1984**
Van de Merwe TJ, Silverstone T, Ankier SI, Warrington SJ, Turner P. A double-blind non-crossover placebo-controlled study between group comparison of trazodone and amitriptyline on cardiovascular function in major depressive disorder. Psychopathology. 1984;17 Suppl 2:64-76.

280. **Vartiainen 1994 (MIR 84023 FDA)**
Vartiainen H, Leinonen E. Double-blind study of mirtazapine and placebo in hospitalized patients with major depression. Eur Neuropsychopharmacol. 1994 Jun;4(2):145-50.
http://digitalcommons.ohsu.edu/fdadrug/20/
https://digitalcollections.ohsu.edu/concern/etds/p8418n49j?locale=en

281. **VEN 600A-303 (FDA)**
http://digitalcommons.ohsu.edu/fdadrug/24/
https://digitalcollections.ohsu.edu/concern/etds/pk02c992m?locale=en

282. **VEN 600A-313 (FDA)**
http://digitalcommons.ohsu.edu/fdadrug/24/
https://digitalcollections.ohsu.edu/concern/etds/pk02c992m?locale=en

283. **VEN XR 367 (FDA)**
http://digitalcommons.ohsu.edu/fdadrug/25/
https://digitalcollections.ohsu.edu/concern/etds/z029p503q?locale=en

284. **Versiani 2000 (Study 091)**
Versiani M, Amin M, Chouinard G. Double-blind, placebo-controlled study with reboxetine in inpatients with severe major depressive disorder. J Clin Psychopharmacol. 2000;20(1):28-34
https://www.iqwig.de/de/projekte-ergebnisse/studieninformationen-zu-reboxetin.3304.html

285. **Wade 2002 (ESC Study 99001 - FDA)**
Wade A, Michael Lemming O, Bang Hedegaard K. Escitalopram 10 mg/day is effective and well tolerated in a placebo-controlled study in depression in primary care. Int Clin Psychopharmacol. 2002;17(3):95-102.
http://www.lundbeck.com/upload/trials/files/pdf/completed/99001_CTRS_final_30Dec200[illegible]pdf

286. **Walczak 1996**
Walczak DD, Apter JT, Halikas JA, Borison RL, Carman JS, Post GL, Patrick R, Cohn JB, Cunningham LA, Rittberg B, Preskorn SH, Kang JS, Wilcox CS. The oral dose-effect relationship for fluvoxamine: a fixed-dose comparison against placebo in depressed outpatients. Ann Clin Psychiatry. 1996 Sep;8(3):139-51.

287. **Wang 2014 (EUCTR2005-005052-40, NCT00351169, D1448C00004)**
Wang G, McIntyre A, Earley WR, Raines SR, Eriksson H. A randomized, double-blind study of the efficacy and tolerability of extended-release quetiapine fumarate (quetiapine XR) monotherapy in patients with major depressive disorder. Neuropsychiatr Dis Treat. 2014 Jan 30;10:201-16.

288. **WELL 029**

http://digitalcommons.ohsu.edu/fdadrug/21/
https://digitalcollections.ohsu.edu/concern/etds/8k71nh40n?locale=en

289. **WELL AK1A4006**
http://digitalcommons.ohsu.edu/fdadrug/21/
https://digitalcollections.ohsu.edu/concern/etds/8k71nh40n?locale=en

290. **Wellbutrin 06**
http://digitalcommons.ohsu.edu/fdadrug/21/
https://digitalcollections.ohsu.edu/concern/etds/8k71nh40n?locale=en

291. **Wellbutrin 25**
http://digitalcommons.ohsu.edu/fdadrug/21/
https://digitalcollections.ohsu.edu/concern/etds/8k71nh40n?locale=en

292. **Wilcox 1994**
Wilcox CS, Cohn JB, Katz BB, Mijares CP, Guarino JJ, Panagides J, DeFrancisco DF. A double-blind, placebo-controlled study comparing mianserin and amitriptyline in moderately depressed outpatients. Int Clin Psychopharmacol. 1994 Winter;9(4):271-9.

293. **Zajecka 2010 (CAGO178A2301, NCT00411099)**
Zajecka J, Schatzberg A, Stahl S. Efficacy and safety of agomelatine in the treatment of major depressive disorder. J Clin Psychopharm 2010; 30: 135-44.
https://clinicaltrials.gov/ct2/show/NCT00411099

294. **Zhang 2014**
Zhang L, Xie WW, Li LH,. Efficacy and safety of prolonged-release trazodone in major depressive disorder. Pharmacology. 2014;94(5-6):199-206.

295. **845**
http://digitalcommons.ohsu.edu/fdadrug/21/
https://digitalcollections.ohsu.edu/concern/etds/8k71nh40n?locale=en

296. **003-008 (FDA)**
http://digitalcommons.ohsu.edu/fdadrug/20/
https://digitalcollections.ohsu.edu/concern/etds/p8418n49j?locale=en

297. **003-042**
http://digitalcommons.ohsu.edu/fdadrug/20/
https://digitalcollections.ohsu.edu/concern/etds/p8418n49j?locale=en

298. **003-048**
http://digitalcommons.ohsu.edu/fdadrug/20/
https://digitalcollections.ohsu.edu/concern/etds/p8418n49j?locale=en

299. **030A2-0004/030A2-0005 (FDA)**
http://digitalcommons.ohsu.edu/fdadrug/23/
https://digitalcollections.ohsu.edu/concern/etds/3484zh30d?locale=en

300. **03A0A-004A (FDA)**
http://digitalcommons.ohsu.edu/fdadrug/23/
https://digitalcollections.ohsu.edu/concern/etds/3484zh30d?locale=en

301. **0600A1-300** Unpublished Report-Wyeth Research Philadelphia: a randomized, double-blind comparison of venlafaxine, amitriptyline, and placebo capsules in inpatients with major depression: final report (Protocol: 0600A1-300-US). 19-11-2002.

302. **0600B1-384**

Unpublished Report-Wyeth-Ayerst USA: A double-blind, placebo-controlled, comparative study of an extended release formulation of venlafaxine and imipramine on the time of onset of anti-depressant response in patients with severe major depression: final report (Protocol 600B1-384-US/EU/CA). 12-9-2002.

303. **29060 07 001**
http://www.gsk-clinicalstudyregister.com/study/29060/07/001#rs

304. **244 (EMD 68 843-009)**
http://www.accessdata.fda.gov/drugsatfda_docs/nda/2011/022567Orig1s000MedR.pdf

305. **245 (EMD 68 843-010)**
http://www.accessdata.fda.gov/drugsatfda_docs/nda/2011/022567Orig1s000MedR.pdf

306. **246 (SB 659746-003)**
http://www.accessdata.fda.gov/drugsatfda_docs/nda/2011/022567Orig1s000MedR.pdf

307. **247 (SB 659746-014)**
http://www.accessdata.fda.gov/drugsatfda_docs/nda/2011/022567Orig1s000MedR.pdf

308. **248 (SB 659746-002)**
http://www.accessdata.fda.gov/drugsatfda_docs/nda/2011/022567Orig1s000MedR.pdf
