# Supplementary material for: Raising Placebo Efficacy in Antidepressant Trials Across Decades Explained by Small-Study Effects: A Meta-Reanalysis
Source: Front Psychiatry. 2020 Jul 28;11:633. doi: 10.3389/fpsyt.2020.00633 (PMC7399231; doi:10.3389/fpsyt.2020.00633)
Supplement: Supplementary file 2 [file DataSheet_2.pdf]

Supplementary Material: Raising placebo efficacy in antidepressant trials across decades explained by small-study effects: a meta-reanalysis

1 DATA SOURCES

Figure. PRISMA flow diagram. The four additional trials (Feiger et al., 2009; Septien-Velez et al., 2007; Liebowitz et al., 2007; Kornstein et al., 2010) provided by Furukawa et al. (Furukawa et al., 2016) are detailed in the **Supplementary List of Identified Studies**.

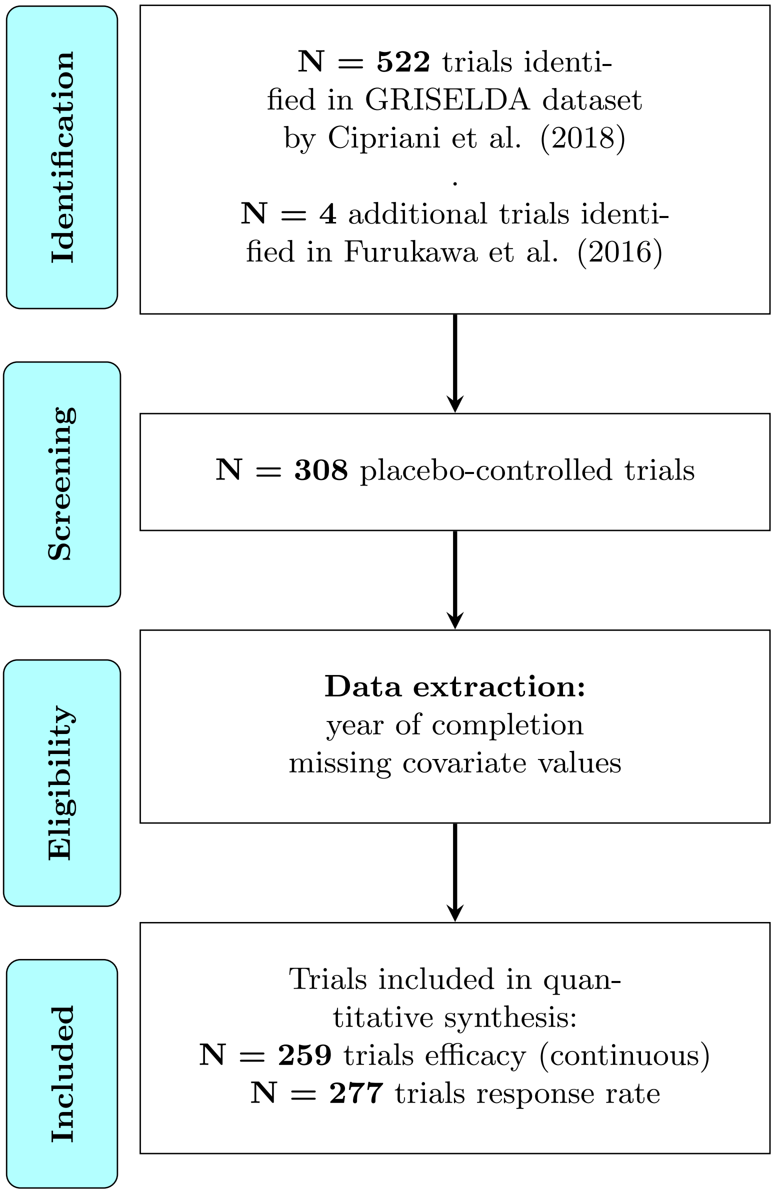

Table . Data extraction. Data were extracted from the literature for missing covariates values on the covariate study center. See the **supplementary List** for details on the studies.

Study ID	Study center
0600B1-384	multi-center
Buchsbaum1997	single-center
Dimidjian2006	multi-center
FDA 244 (EMD 68 843-009)	multi-center
FDA 245 (EMD 68 843-010)	multi-center
FDA 246 (SB 659746-003)	multi-center
FDA 247 (SB 659746-014)	multi-center
FDA 248 (SB 659746-002)	multi-center
Fabre1979	single-center
Fabre1995	multi-center
Feighner1980	single-center
Feighner1984	single-center
Feighner1989b	single-center
Georgotas1982	single-center
Gerner1980	single-center
Halikas1995 (MIR 003-023 - FDA)	single-center
Hormazabal1985	single-center
Itil1983	single-center
Katz1993a	multi-center
Katz1993b	multi-center
Kusalic1993	single-center
Larsen1989	multi-center
MIR 003-003 (FDA)	single-center
MIR 003-020 (FDA)	single-center
MIR 003-021 (FDA)	single-center
NKD20006 (NCT00048204)	multi-center
Olie1997	multi-center
PZ/109	multi-center
PZ/111	multi-center
SCT-MD-49 (NCT00668525)	multi-center
Smith1990 (MIR 003-024 FDA)	single-center
Study 25 (FDA) (Rickels1986)	single-center
VEN 600A-313 (FDA)	multi-center

Figure. Covariate distribution study year. Histograms illustrating the distribution of study year across studies.

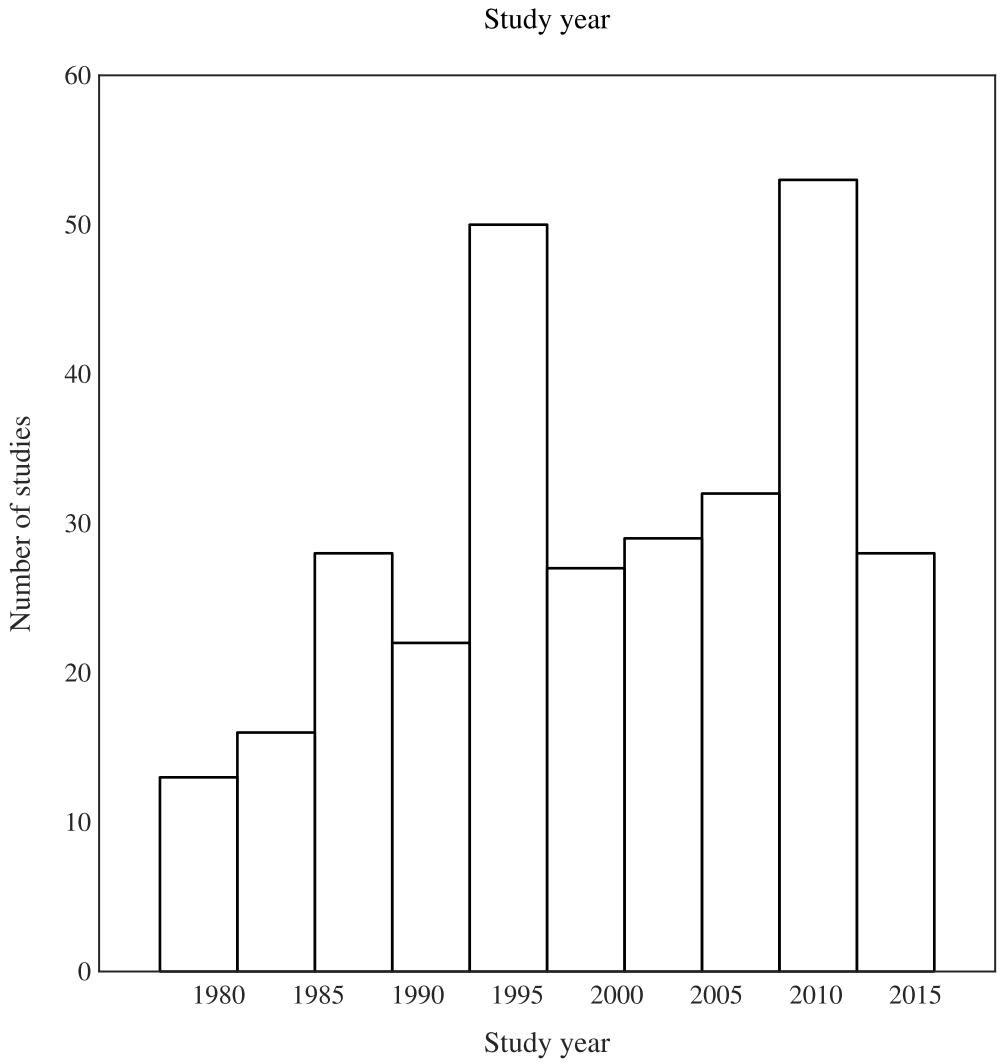

Figure. Covariate distribution trial-level. Histograms illustrating the distributions of the trial-level covariates.

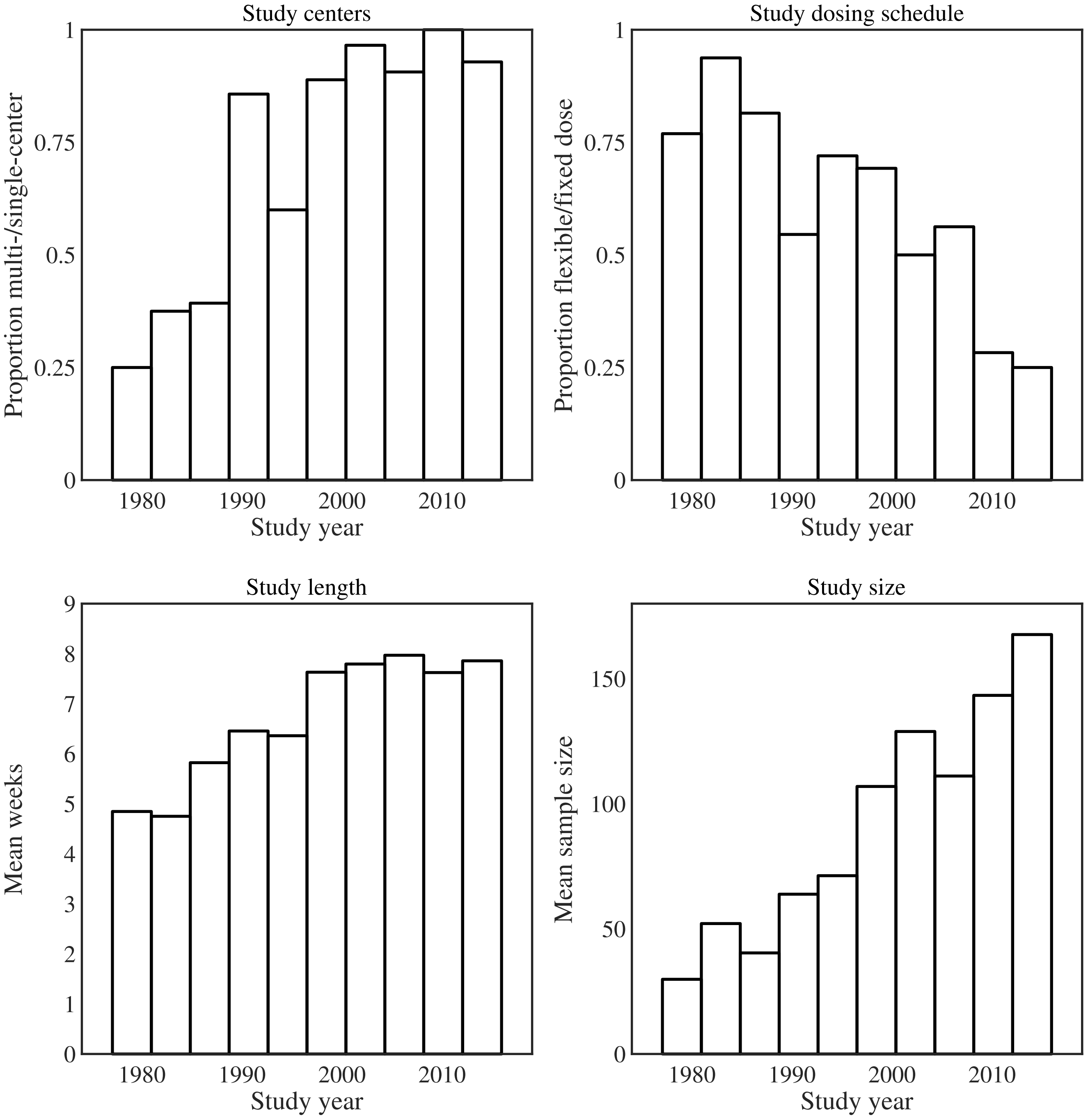

2 STRUCTURAL BREAK ANALYSIS

Table . Structural breaks. Structural break analysis was conducted using the `breakpoints` command in the `strucchange` package (Zeileis, 2019) in R. Reported are the observed break dates, F-statistics, and corresponding p-values obtained from the Chow-test (supF test) (Andrews, 1993; Andrews and Ploberger, 1994). No break date was observed for efficacy (continuous).

	Break dates	F-stats	p-val
Efficacy (cont.)	-	3.09	0.139
Response	1990	11.45	0.000

Figure. Structural breaks. Plots illustrating potential breaks of placebo effect sizes across study year. Vertical dashed lines indicate the break dates, horizontal red lines indicate the confidence intervals. No break date was observed for efficacy (continuous).

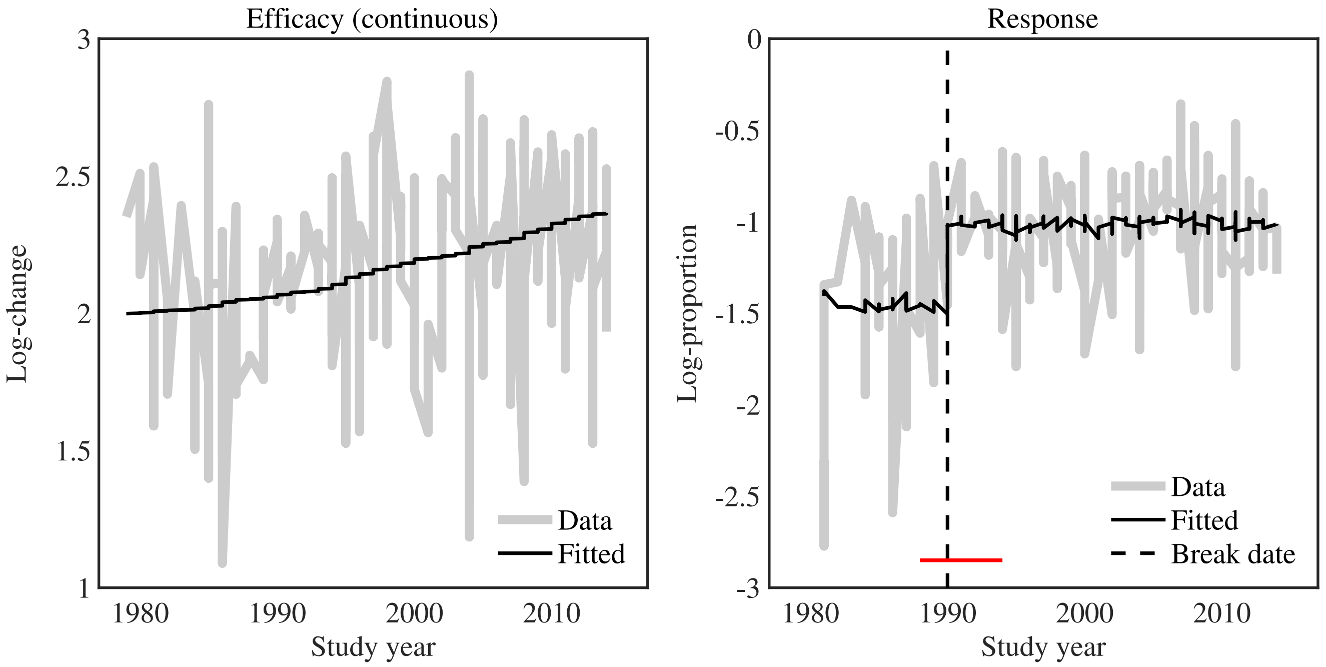

Figure. Structural breaks F-statistics. Plots illustrating the F-statistics of potential break dates of placebo effect sizes across study year. Horizontal red lines indicate the boundaries of the F-statistics. No break date was observed for efficacy (continuous).

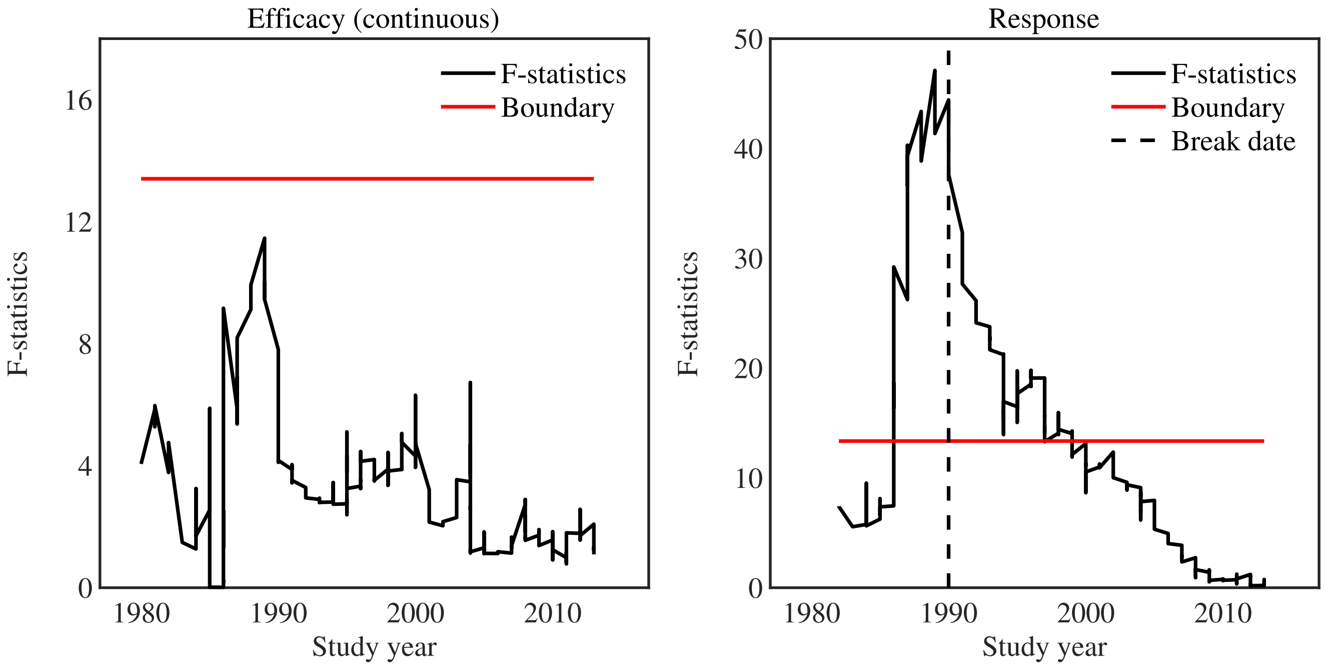

3 RESTRICTED CUBIC SPLINES (RCS)

Table . RCS knot locations. Listed are results of a sensitivity analysis examining different knot locations of restricted cubic spline (RCS). RCS were examined using 3 knots by varying the 1st and 3rd knots, while the middle knots were set at the break date (1990). RCS comparison was based on the corrected Akaike information criterion (AICc) as an estimator of model fit. Smaller values are assumed to represent a better fit. Since the differences between AICs were marginal and did not affect overall conclusions, AICc was not used for knot selection.

Knots	Efficacy (cont.) AICc	Response AICc
1980,1990,1991	33.99	33.54
1980,1990,1996	33.54	32.64
1980,1990,2002	33.44	31.82
1980,1990,2008	33.63	30.68
1980,1990,2013	33.75	30.10
1982,1990,1991	33.96	33.47
1982,1990,1996	33.52	32.42
1982,1990,2002	33.43	31.78
1982,1990,2008	33.63	30.64
1982,1990,2013	33.75	30.06
1984,1990,1991	33.91	33.34
1984,1990,1996	33.48	32.29
1984,1990,2002	33.42	31.50
1984,1990,2008	33.62	30.58
1984,1990,2013	33.75	30.00
1987,1990,1991	33.75	32.97
1987,1990,1996	33.56	32.00
1987,1990,2002	33.55	31.32
1987,1990,2008	33.62	30.43
1987,1990,2013	33.76	29.60
1989,1990,1991	33.77	32.67
1989,1990,1996	33.47	31.80
1989,1990,2002	33.53	31.20
1989,1990,2008	33.63	30.35
1989,1990,2013	33.77	29.51

4 LIMIT META-ANALYSIS

Table . Limit meta-analysis heterogeneity statistics. Listed are heterogeneity statistics of the limit meta-analysis quantifying SSE in terms of residual heterogeneity (Q_E), heterogeneity accounted for SSE (Q_{SSE}), τ^2, I^2, and heterogeneity that remains after SSE are accounted for (G^2, range 0 - 100%) (Rücker et al., 2011, 2010).

	Q_E	Q_{SSE}	τ^2	I^2	G^2
Efficacy (cont.)	1480	638	0.229	88%	3%
Response	666	390	0.209	74%	24%

Table . Limit meta-analysis test statistics. Listed are statistics of the limit meta-analysis. The parameter α corresponds to the intercept, the bias introduced by small-study effects, interpreted as the expected shift in the standardized treatment-effect estimate for a hypothetical 'small' study with zero precision. Its slope (parameter β) represents the treatment-effect estimate of the extended model allowing for small-study effects (Rücker et al., 2011, 2010).

	z	p-value	bias (intercept,α)	slope (β)
Efficacy (cont.)	-486.88	0.000	3.30	-3.08
Response	-110.08	0.000	-2.95	-0.25

Table . Egger test statistics. Listed are statistics of a Egger test assessing funnel plot asymmetry conducted using the `metabias` command in the `meta` package (Schwarzer, 2019), for comparison with the limit meta-analysis.

	t	p-value	bias (intercept)	slope
Efficacy (cont.)	10.50	0.000	3.57	-2.59
Response	-12.70	0.000	-2.83	-0.60

Figure. Limit meta-analysis funnel plots. Funnel plots visualizing the adjustment (shrinkage) process of the limit meta-analysis, based on the extended random-effects model constructed using the standard error as the measure of accuracy. The original trial estimates (black circles) move parallel to the x-axis (red lines) to the adjusted shrunken trial estimates (white circles) towards the new center of the funnel plot (Rücker et al., 2011, 2010). The original and adjusted shrunken trial estimates are relatively far from each other and the red lines representing the adjustment span large proportions of the x-axis, thus suggesting significant SSE.

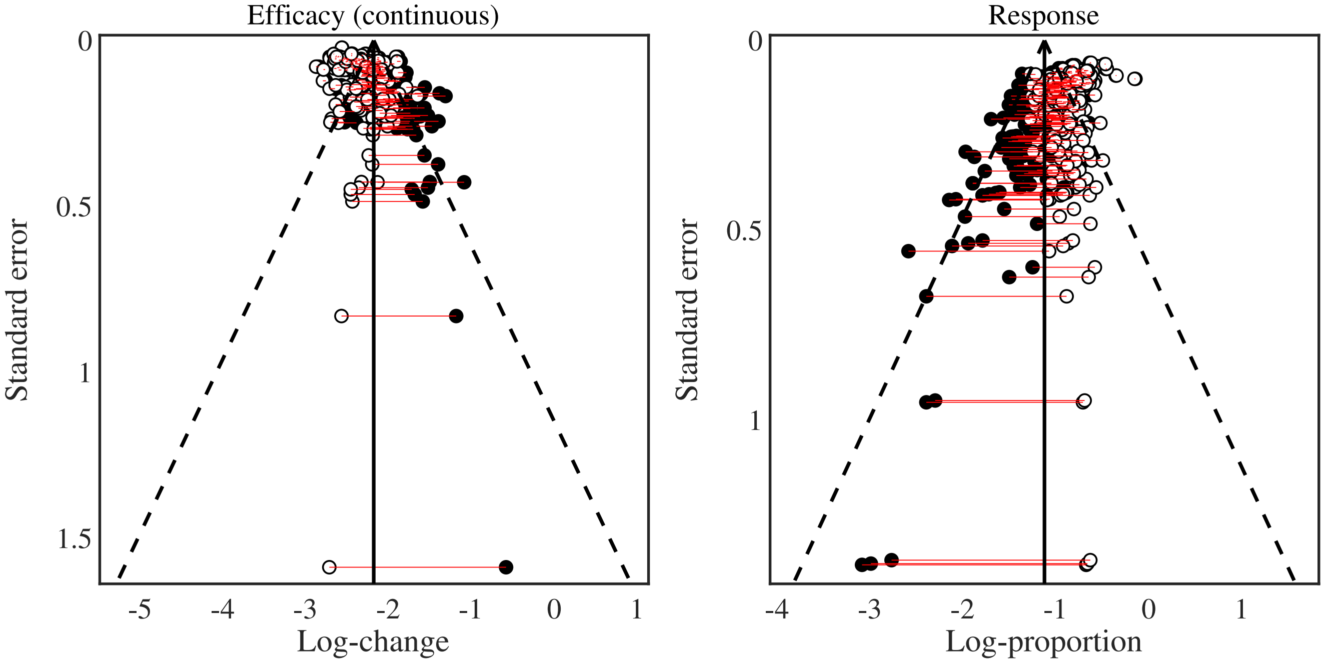

Figure. Limit meta-analysis radial plots. Radial plots as proposed by Rucker et al. (Rücker et al., 2011, 2010) visualizing the adjustment (shrinkage) process of the limit meta-analysis. The red regression lines are derived from the original (black circles) and the adjusted shrunken (white circles) trial estimates. For the shrunken estimates the slope (β) represents the treatment-effect estimate of the extended random model allowing for SSE, and the intercept (α) corresponds to the bias introduced by SSE. For the binary outcomes, the slopes of the original and shrunken regression lines differ more compared to the continuous outcomes, thus suggesting larger SSE. The farer away the dots in the radial plots lie from the regression line, the more variation is not explained by SSE, as indicated by heterogeneity measured by G^2.

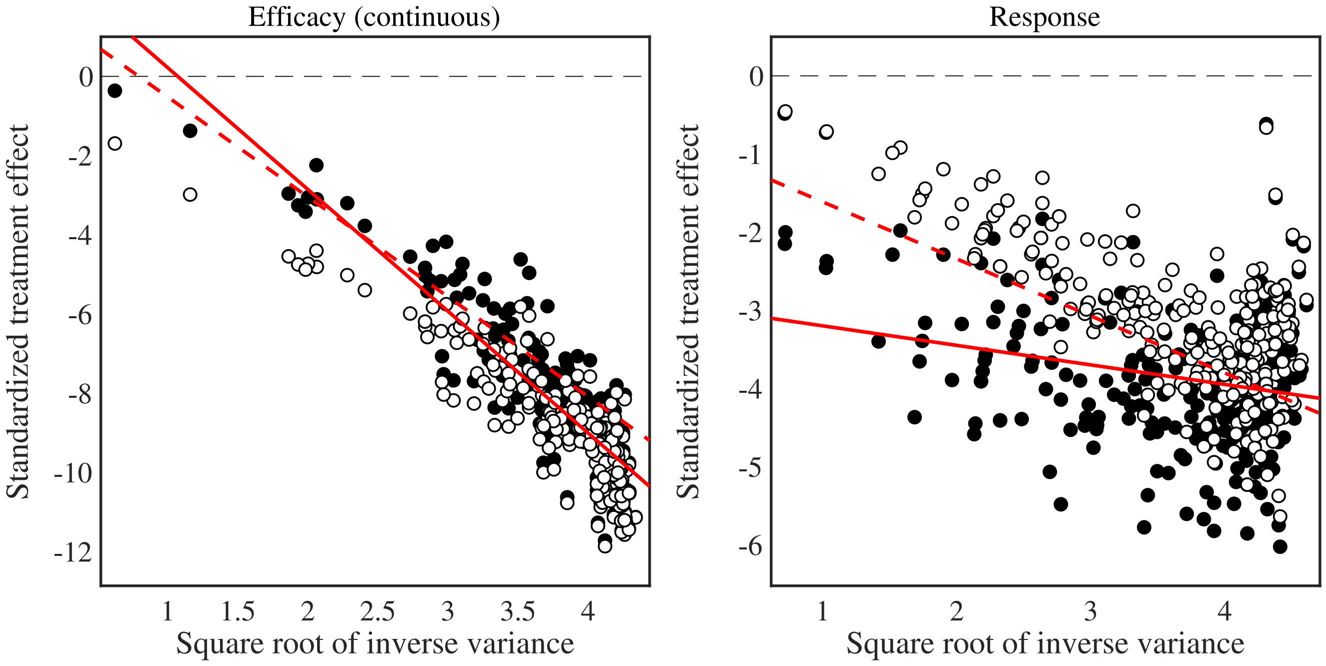

Table . Meta-analytical power. To visualize SSE, the power of individual studies was computed based on its standard errors using a two-sided Wald test as implemented in the `metaviz` package (Kossmeier, 2019). The power was graphically illustrated assuming high power (100%), moderate power (100-80%), and low power (<80%). In addition, a test for excess significance (Ioannidis and Trikalinos, 2007) was conducted which evaluates whether the number of formally significant trials among studies is too large based on the power that these studies have to detect plausible effects (α = 0.05). The test itself does not consider SSE but can have similar sources. Together, this allows to examine whether underpowered but significant studies are present, and to visually assess if there is an excess of low-powered significant effects (Ioannidis and Trikalinos, 2007). Results are reported in terms of a) the median power of all studies and the percentages of studies with high (100%), moderate (100-80%), and low power (<80%), b) the true effect size necessary such that the median power of the studies would have been 33% or 66%, c) p-values of the test of excess significance (Ioannidis and Trikalinos, 2007), and d) the R-Index for expected replicability (Schimmack, 2016).

	Efficacy (cont.)	Response
Median power	100%	100%
High power (100%)	91%	17%
Adequate power (100-80%)	8%	70%
Low power (<80%)	0.4%	13%
True effect 33%	0.13	0.20
True effect 66%	0.21	0.32
Excess significance (p-value)	0.236	0.000
R-index	100%	100%

Figure. Power-enhanced funnel plots. Power-enhanced funnel plots created following the command `viz_sunset` in the `metaviz` package (Kossmeier, 2019). The funnel plots display the power of individual studies to detect an effect of interest using a two-sided Wald test.

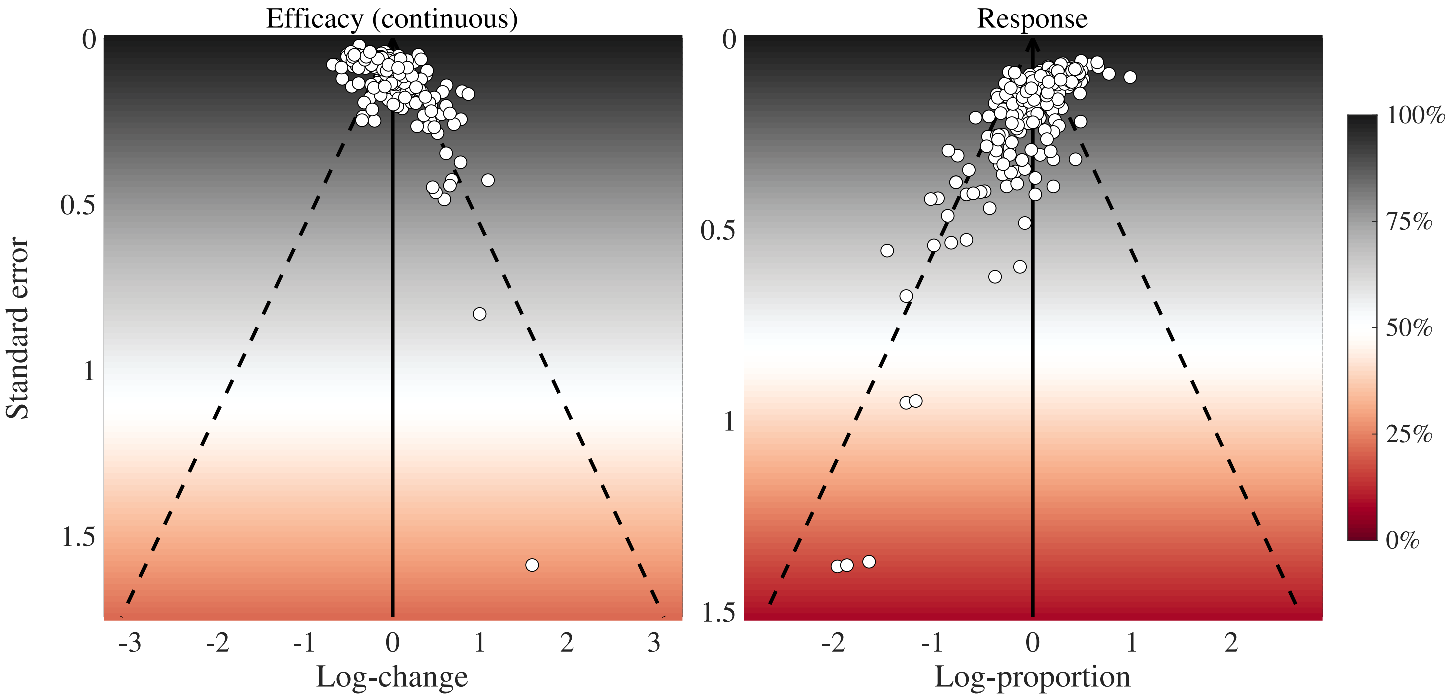

5 MULTIMODEL INFERENCE

Multimodel inference was conducted using the `glmulti` package (Calcagno, 2019), which provides the functionality for multimodel averaging using an information-theoretic approach. An extensive model comparison was conducted considering all possible covariate combinations. Multimodel inference was performed both before and after adjustment for SSE based on the limit meta-analysis. The only constraint was that the nonlinear component of study year was only included in the presence of the linear component, since the former alone is uninterpretable. Together this resulted in N = 47 models. Models were fitted using the `rma` function in the `metafor` package (Viechtbauer, 2010). All procedures followed common guidelines (Grueber et al., 2011).

Continuous covariates (study year, study length, study size) were standardized by centering at the mean and dividing by the standard deviation, because standardization provides effect sizes that are on comparable scales when derived from covariates with different units (Grueber et al., 2011):

$$x_i = (\text{value}_i - \text{value of centralization})/\text{standard deviation} \qquad \text{(Eq. S1)}$$

Categorical covariates (study center, study dosing schedule) were coded as follows:

$$x_i = \begin{cases} 1, & \text{if trial i was a multi-center/flexible dose trial} \\ 0, & \text{if trial i was a single-center/fixed dose trial} \end{cases} \qquad \text{(Eq. S2)}$$

Multimodel weighting was based on the corrected Akaike information criterion (AICc). Each model is assigned a relative evidence weight, which is computed as exp(-ΔAICc/2), where ΔAICc is the difference in AICc between a model and the best model. The weights are normalized so that they sum up to one across all models. The weights can thus be interpreted as probabilities for each model to be the best in the set (Calcagno, 2019). The top model sets for each outcome, defined as the top-ranked models summing up to 95% evidence weight (Grueber et al., 2011; Burnham and Anderson, 2002), were used for multimodel averaging.

Multimodel averaging was used to derived unconditional covariate estimates across the top model sets for each outcome (Calcagno, 2019). The model-averaged covariate estimates are weighted averages of the model coefficients across the various models (with weights equal to the AICs evidence weights). The estimates are called 'unconditional' as they are not conditional on any one model. Similarly, multimodel predictions and corresponding confidence intervals (95% CI) were computed across the top model sets (Calcagno, 2019) at the means of all covariates.

Multimodel heterogeneity statistics, estimated based on the method of moments (DerSimonian and Laird, 1986), were also computed based on the top model sets (Calcagno, 2019) and reported in terms of τ^2 and I^2, weighted based on a custom code derived from `glmulti` (Calcagno, 2019).

5.1 Multimodel comparison

Table . Multimodel comparison: efficacy (cont.). Listed are all models (total N = 47) assessing the relative covariate importance on placebo. Models are ranked based on the corrected Akaike information criterion (AICc), together with the corresponding normalized relative evidence weights (W). Shown are also heterogeneity statistics in terms τ^2 and I^2. The top model set summing up to 95% evidence weight is highlighted (red). Y1/Y2 = study year linear/nonlinear.

	AICc	W	τ^2	I^2
yi ∼ 1 + Y1 + Size	-77	0.221	0.028	82%
yi ∼ 1 + Y1	-75	0.096	0.028	82%
yi ∼ 1 + Y1 + Dosing + Size	-75	0.087	0.028	82%
yi ∼ 1 + Y1 + Length + Size	-75	0.081	0.028	82%
yi ∼ 1 + Y1 + Y2 + Size	-75	0.063	0.028	82%
yi ∼ 1 + Y1 + Dosing	-74	0.051	0.028	82%
yi ∼ 1 + Y1 + Length	-74	0.043	0.028	82%
yi ∼ 1 + Y1 + Center + Size	-73	0.036	0.028	82%
yi ∼ 1 + Y1 + Dosing + Length + Size	-73	0.033	0.028	82%
yi ∼ 1 + Y1 + Y2 + Dosing + Size	-73	0.025	0.028	82%
yi ∼ 1 + Y1 + Y2	-73	0.024	0.028	82%
yi ∼ 1 + Y1 + Dosing + Length	-73	0.024	0.028	82%
yi ∼ 1 + Y1 + Y2 + Length + Size	-73	0.022	0.028	82%
yi ∼ 1 + Dosing	-72	0.017	0.030	83%
yi ∼ 1 + Y1 + Center + Dosing + Size	-72	0.017	0.028	82%
yi ∼ 1 + Y1 + Center	-72	0.014	0.029	82%
yi ∼ 1 + Y1 + Center + Length + Size	-72	0.013	0.028	82%
yi ∼ 1 + Y1 + Y2 + Dosing	-71	0.012	0.028	82%
yi ∼ 1 + Y1 + Y2 + Center + Size	-71	0.010	0.028	82%
yi ∼ 1 + Y1 + Y2 + Length	-71	0.010	0.028	82%
yi ∼ 1 + Y1 + Y2 + Dosing + Length + Size	-71	0.009	0.028	82%
yi ∼ 1 + Y1 + Center + Dosing	-71	0.008	0.029	82%
yi ∼ 1 + Size	-70	0.008	0.030	83%
yi ∼ 1 + Y1 + Center + Dosing + Length + Size	-70	0.007	0.028	82%
yi ∼ 1 + Length	-70	0.007	0.030	83%
yi ∼ 1 + Y1 + Center + Length	-70	0.006	0.029	82%
yi ∼ 1 + Dosing + Size	-70	0.006	0.030	83%
yi ∼ 1 + Y1 + Y2 + Dosing + Length	-70	0.005	0.028	82%
yi ∼ 1 + Y1 + Y2 + Center + Dosing + Size	-70	0.005	0.028	82%
yi ∼ 1 + Center	-69	0.005	0.030	83%
yi ∼ 1 + Dosing + Length	-69	0.004	0.030	83%
yi ∼ 1 + Center + Dosing	-69	0.004	0.030	83%
yi ∼ 1 + Y1 + Center + Dosing + Length	-69	0.004	0.029	82%
yi ∼ 1 + Y1 + Y2 + Center + Length + Size	-69	0.004	0.028	82%
yi ∼ 1 + Y1 + Y2 + Center	-69	0.003	0.029	82%
yi ∼ 1 + Length + Size	-68	0.002	0.030	83%
yi ∼ 1 + Center + Size	-68	0.002	0.030	83%
yi ∼ 1 + Y1 + Y2 + Center + Dosing + Length + Size	-68	0.002	0.028	82%
yi ∼ 1 + Y1 + Y2 + Center + Dosing	-68	0.002	0.029	82%
yi ∼ 1 + Center + Dosing + Size	-67	0.002	0.030	83%
yi ∼ 1 + Dosing + Length + Size	-67	0.002	0.030	83%
yi ∼ 1 + Y1 + Y2 + Center + Length	-67	0.001	0.029	82%
yi ∼ 1 + Center + Length	-67	0.001	0.030	83%
yi ∼ 1 + Center + Dosing + Length	-66	0.001	0.030	83%
yi ∼ 1 + Y1 + Y2 + Center + Dosing + Length	-66	0.001	0.029	82%
yi ∼ 1 + Center + Length + Size	-65	0.001	0.030	83%
yi ∼ 1 + Center + Dosing + Length + Size	-65	0.000	0.030	83%

Table . Multimodel comparison: response. Listed are all models (total N = 47) assessing the relative covariate importance on placebo. Models are ranked based on the corrected Akaike information criterion (AICc), together with the corresponding normalized relative evidence weights (W). Shown are also heterogeneity statistics in terms τ^2 and I^2. The top model set summing up to 95% evidence weight is highlighted (red). Y1/Y2 = study year linear/nonlinear.

	AICc	W	τ^2	I^2
yi ~ 1 + Y1 + Y2 + Length + Size	-120	0.191	0.014	46%
yi ~ 1 + Length + Size	-119	0.132	0.014	48%
yi ~ 1 + Y1 + Y2 + Size	-119	0.112	0.014	47%
yi ~ 1 + Y1 + Length + Size	-119	0.103	0.014	47%
yi ~ 1 + Y1 + Y2 + Center + Length + Size	-118	0.082	0.014	47%
yi ~ 1 + Center + Length + Size	-117	0.052	0.015	48%
yi ~ 1 + Y1 + Y2 + Center + Size	-117	0.051	0.014	47%
yi ~ 1 + Y1 + Y2 + Dosing + Length + Size	-117	0.044	0.014	46%
yi ~ 1 + Y1 + Center + Length + Size	-117	0.037	0.014	48%
yi ~ 1 + Dosing + Length + Size	-117	0.033	0.014	48%
yi ~ 1 + Y1 + Dosing + Length + Size	-116	0.032	0.014	47%
yi ~ 1 + Y1 + Y2 + Dosing + Size	-116	0.027	0.014	47%
yi ~ 1 + Y1 + Size	-115	0.019	0.015	48%
yi ~ 1 + Y1 + Y2 + Center + Dosing + Length + Size	-115	0.018	0.014	47%
yi ~ 1 + Center + Dosing + Length + Size	-115	0.012	0.015	48%
yi ~ 1 + Y1 + Y2 + Center + Dosing + Size	-115	0.012	0.014	47%
yi ~ 1 + Y1 + Center + Dosing + Length + Size	-114	0.012	0.014	48%
yi ~ 1 + Y1 + Dosing + Size	-114	0.007	0.015	48%
yi ~ 1 + Y1 + Center + Size	-114	0.007	0.015	49%
yi ~ 1 + Size	-113	0.007	0.015	49%
yi ~ 1 + Center + Size	-112	0.004	0.015	50%
yi ~ 1 + Y1 + Center + Dosing + Size	-112	0.003	0.015	49%
yi ~ 1 + Dosing + Size	-110	0.002	0.015	49%
yi ~ 1 + Center + Dosing + Size	-109	0.001	0.015	50%
yi ~ 1 + Y1 + Y2 + Center	-98	0.000	0.016	51%
yi ~ 1 + Y1 + Y2	-98	0.000	0.016	51%
yi ~ 1 + Y1 + Y2 + Center + Length	-97	0.000	0.016	51%
yi ~ 1 + Y1 + Y2 + Length	-97	0.000	0.016	51%
yi ~ 1 + Center + Length	-95	0.000	0.017	52%
yi ~ 1 + Length	-95	0.000	0.017	52%
yi ~ 1 + Center	-95	0.000	0.017	53%
yi ~ 1 + Y1 + Y2 + Center + Dosing	-95	0.000	0.016	51%
yi ~ 1 + Y1 + Y2 + Dosing	-94	0.000	0.017	51%
yi ~ 1 + Y1 + Length	-94	0.000	0.017	52%
yi ~ 1 + Y1 + Y2 + Center + Dosing + Length	-94	0.000	0.016	51%
yi ~ 1 + Y1 + Center + Length	-94	0.000	0.017	52%
yi ~ 1 + Y1	-93	0.000	0.018	53%
yi ~ 1 + Y1 + Y2 + Dosing + Length	-93	0.000	0.017	51%
yi ~ 1 + Dosing	-93	0.000	0.018	53%
yi ~ 1 + Y1 + Center	-93	0.000	0.017	53%
yi ~ 1 + Center + Dosing + Length	-92	0.000	0.017	52%
yi ~ 1 + Dosing + Length	-92	0.000	0.017	52%
yi ~ 1 + Center + Dosing	-92	0.000	0.018	53%
yi ~ 1 + Y1 + Dosing + Length	-91	0.000	0.017	52%
yi ~ 1 + Y1 + Dosing	-91	0.000	0.018	53%
yi ~ 1 + Y1 + Center + Dosing + Length	-90	0.000	0.017	52%
yi ~ 1 + Y1 + Center + Dosing	-90	0.000	0.017	53%

Figure. Multimodel covariate importance. Bar plots illustrating the relative evidence weights of the covariates across the top model sets. The vertical red lines are drawn at 0·8 AICc weights, which can be used as cutoff to differentiate between important and not so important variables, but this is an arbitrary division (Calcagno, 2019; Anderson, 2007). Y1/Y2 = study year linear/nonlinear.

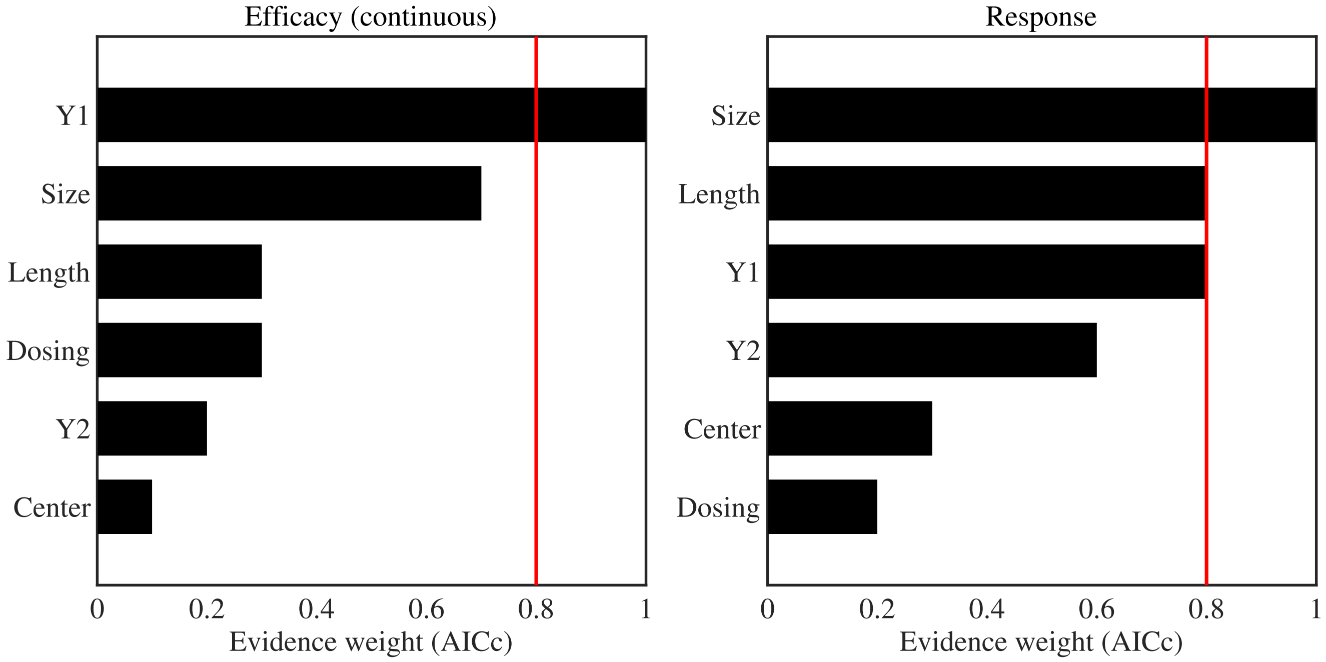

Figure. Multimodel AICc profile. Plots illustrating the AICc profile of the best to worst model. The horizontal red lines differentiate between models whose AICc value is less versus more than 2 units away from that of the best model (i.e., the model with the lowest AICc). Sometimes this is taken as a cutoff, so that models with values more than 2 units away are considered substantially less plausible than those with AICc values closer to that of the best model; however, this is again an arbitrary division (Anderson, 2007; Calcagno, 2019). Highlighted (red) are the AICc of the model used by Furukawa et al. (Furukawa et al., 2016), i.e., yi $\sim$ 1 + Year linear + Center + Dosing + Length + Size.

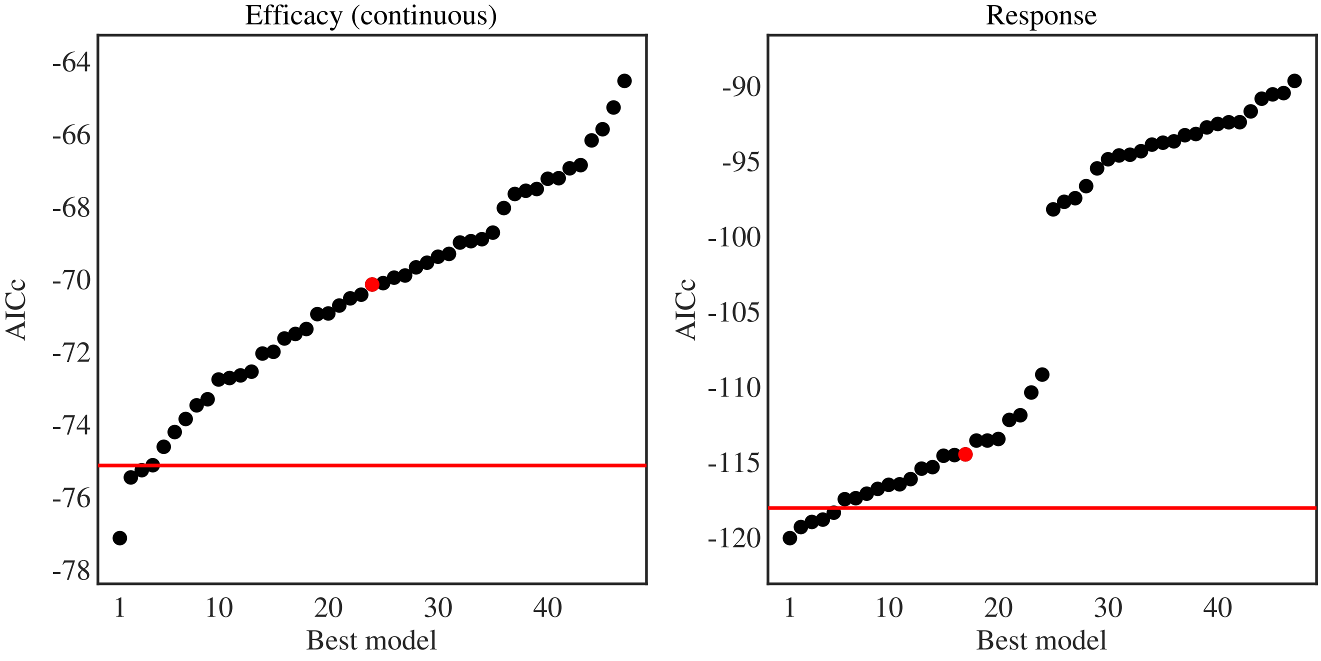

Figure. Multimodel evidence weight profile. Plots illustrating the normalized evidence weight profile based on the AICc of the best to worst model. The vertical red lines delineate models that sum up to 95%; this cutoff was used in the present analysis to define the top model sets. Highlighted (red) are the evidence weights of the model used by Furukawa et al. (Furukawa et al., 2016), i.e., yi ∼ 1 + Year linear + Center + Dosing + Length + Size.

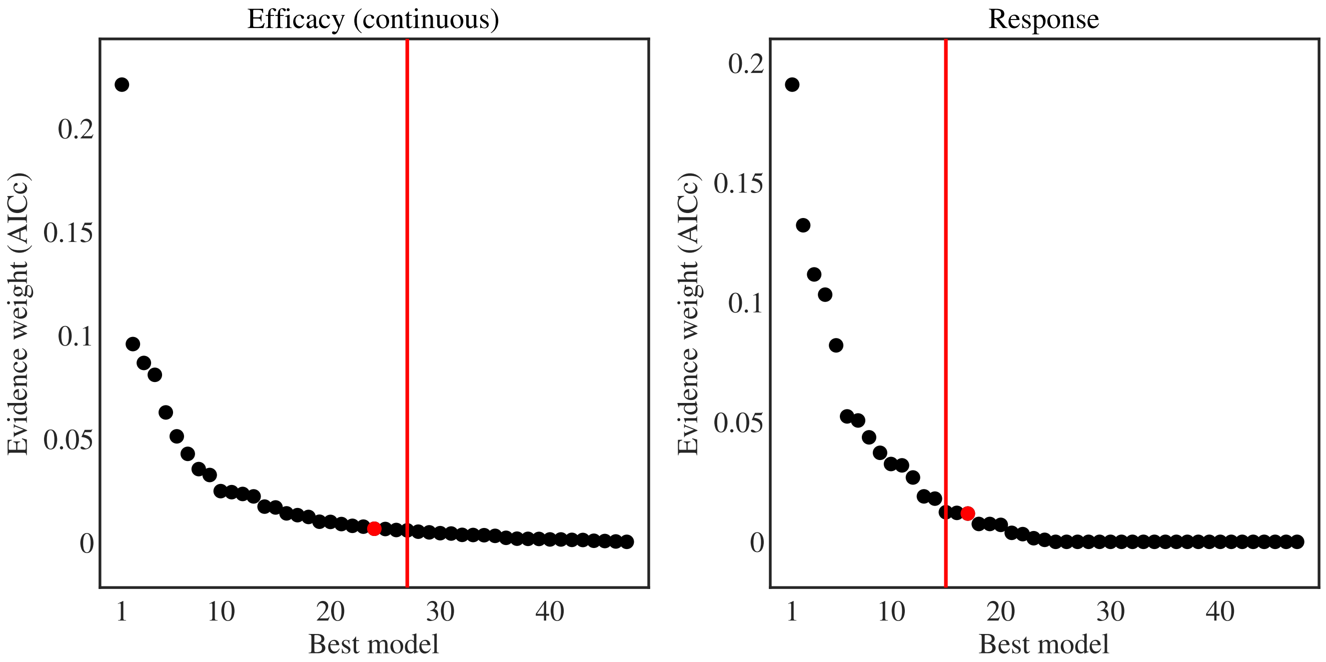

REFERENCES

Feiger AD, Tourian KA, Rosas GR, Padmanabhan SK. A Placebo-Controlled Study Evaluating the Efficacy and Safety of Flexible-Dose Desvenlafaxine Treatment in Outpatients with Major Depressive Disorder. *CNS Spectrums* **14** (2009) 41–50. doi:10.1017/S1092852900020046.

Septien-Velez L, Pitrosky B, Padmanabhan SK, Germain JM, Tourian KA. A randomized, double-blind, placebo-controlled trial of desvenlafaxine succinate in the treatment of major depressive disorder. *International Clinical Psychopharmacology* **22** (2007).

Liebowitz MR, Yeung P, Entsuah R. A Randomized, Double-Blind, Placebo-Controlled Trial of Desvenlafaxine Succinate in Adult Outpatients With Major Depressive Disorder. *The Journal of Clinical Psychiatry* **68** (2007) 1663–1672.

Kornstein SG, Jiang Q, Reddy S, Musgnung J, Guico-Pabia C. Short-Term Efficacy and Safety of Desvenlafaxine in a Randomized, Placebo-Controlled Study of Perimenopausal and Postmenopausal Women With Major Depressive Disorder. *The Journal of Clinical Psychiatry* **71** (2010) 1088–1096.

Furukawa TA, Cipriani A, Atkinson LZ, Leucht S, Ogawa Y, Takeshima N, et al. Placebo response rates in antidepressant trials: A systematic review of published and unpublished double-blind randomised controlled studies. *The Lancet Psychiatry* **3** (2016) 1059–1066. doi:10.1016/S2215-0366(16)30307-8.

[Dataset] Zeileis A. Package ”strucchange” (2019).

Andrews DWK. Tests for Parameter Instability and Structural Change With Unknown Change Point. *Econometrica* **61** (1993) 821–856. doi:10.2307/2951764.

Andrews DWK, Ploberger W. Optimal Tests when a Nuisance Parameter is Present Only Under the Alternative. *Econometrica* **62** (1994) 1383–1414. doi:10.2307/2951753.

Rücker G, Carpenter JR, Schwarzer G. Detecting and adjusting for small-study effects in meta-analysis. *Biometrical Journal* **53** (2011) 351–368. doi:10.1002/bimj.201000151.

Rücker G, Schwarzer G, Carpenter JR, Binder H, Schumacher M. Treatment-effect estimates adjusted for small-study effects via a limit meta-analysis. *Biostatistics* **12** (2010) 122–142. doi:10.1093/biostatistics/kxq046.

[Dataset] Schwarzer G. Package ’meta’ (2019).

[Dataset] Kossmeier M. Package ’metaviz’ (2019).

Ioannidis JP, Trikalinos TA. An exploratory test for an excess of significant findings. *Clinical Trials* **4** (2007) 245–253. doi:10.1177/1740774507079441.

[Dataset] Schimmack U. The replicability-index: Quantifying statistical research integrity. https://replicationindex.wordpress.com/2016/01/31/a-revised-introduction-to-the-r-index/ (2016).

[Dataset] Calcagno V. Package ’glmulti’ (2019).

Viechtbauer W. Conducting Meta-Analyses in R with the metafor Package. *Journal of Statistical Software* **36** (2010) 1–48.

Grueber CE, Nakagawa S, Laws RJ, Jamieson IG. Multimodel inference in ecology and evolution: Challenges and solutions. *Journal of Evolutionary Biology* **24** (2011) 699–711. doi:10.1111/j.1420-9101.2010.02210.x.

Burnham K, Anderson D. *Model Selection and Multimodel Inference:A Practical Information-Theoretic Approach.* (New York: Springer New York) (2002).

DerSimonian R, Laird N. Meta-analysis in clinical trials. *Controlled Clinical Trials* **7** (1986) 177–188. doi:10.1016/0197-2456(86)90046-2.

Anderson D. *Model Based Inference in the Life Sciences: A Primer on Evidence* (New York: Springer) (2007).
